# A Combined Experimental-Theoretical Approach Unveils the Comprehensive Asn Deamidation Kinetics

**DOI:** 10.1021/jacs.6c11010

**Published:** 2026-07-28

**Authors:** Maria Laura De Sciscio, Rosanna Mosetti, Annalisa D’Arco, Stefano Lupi, Mariana Gallo, Thelma A. Pertinhez, Fabrizio Vetica, Mauro Giustini, Marco D’Abramo

**Affiliations:** † Department of Chemistry, University of Rome, Sapienza, P.le A. Moro 5 00185 Rome, Italy; ‡ Physics Department, University of Rome Sapienza, P.le Aldo Moro 5 00185 Rome, Italy; § Laboratory of Biochemistry and Metabolomics, Department of Medicine and Surgery, 9370 University of Parma 43125 Parma, Italy

## Abstract

The mechanism of the spontaneous deamidation reaction of the Asn residue in solution has been disclosed through a combination of experimental and computational methods. Utilizing the dipeptide Ace-AsnGly-NH_2_ (AsnGly) as a model system, the free energy barriers of the reaction steps have been modeled in parallel with time-resolved measurements of all species involved, obtained by NMR and IR spectroscopy. The convergent results of the theoretical and experimental data provide a quantitative description of all the kinetic constants for this four-step reaction. Furthermore, this study lends support to the two-step mechanism hypothesis for succinimide formation proposed in the literature and untangles the molecular origin of the reaction’s pH dependence. By bridging atomic structure to reaction kinetics, this work paves the way for the next-generation prediction algorithms, which are essential for the advancement of biologics.

## Graphical abstract

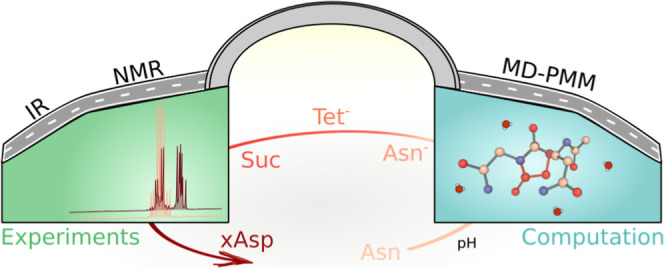

## Introduction

Deamidation of the asparaginyl residue is one of the most common pathways in protein degradation and a harmful critical quality attribute (CQA) in the biotherapeutic products industry.^1^ The need for information at the protein level has driven the research over the past few years; however, progress has been hampered by the lack of a complete understanding of reaction kinetics. A broad range of factors affects deamidation, including the nature of neighboring amino acids and the protein environment.^2–5^ Deamidation rates in oligopeptides are analogous to those observed for amides in unstructured regions of proteins,^4,6,7^ thereby facilitating experimental investigations into the reaction kinetics.

In a previous work,^8^ the deamidation reaction mechanism was investigated on a model system by applying a theoretical-computational approach, which was validated against the available experimental data in similar systems. As established by previous studies,^4,8–14^ the two-step succinimide formation was identified as the rate-determining step, thus implying that all the steps preceding the formation of succinimide may affect the overall kinetics of the reaction. These steps include the deprotonation of the N–H amide of the (*n*+1) residue following Asn, the subsequent attack of the negatively charged amide N (Asn^–^) on the electrophilic side chain carbon amide, forming the tetrahedral intermediate (Tet^–^), and the release of ammonia. The latter, called the deamination step, leads to succinimide (Suc), whose alkaline hydrolysis^8^ finally provides the two isomeric products of the reaction, Asp and *iso*Asp, which we refer collectively to as xAsp.

Succinimide hydrolysis to xAsp has been previously described as a reversible equilibrium between Suc, Asp and *iso*Asp. However, once the products are formed, the formation of Suc is significantly slower than its hydrolysis,^10,15,16^ thus allowing us to approximate this step as an irreversible step for the purpose of this work. The deamidation reaction mechanism is shown in Figure 1A and can be schematically represented as follows

1 $$Asn\;\overset{K}{\to }\;\text{As}n^{-}\overset{k_{1}}{\;\underset{k_{-1}}{⇌}}Tet^{-}\overset{k_{2}\;}{\to }Suc+NH_{3}\;\overset{k_{3}}{\to }\;\left(Asp+isoAsp\right)$$

**Figure 1:**
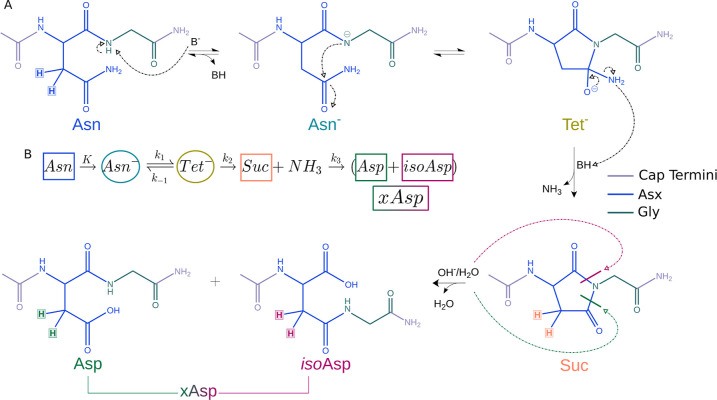
(A) Deamidation proposed mechanism at neutral-to-basic pH. In each 2D structure, the fragment associated with the cap termini, the Asn residue, and the Gly residue are colored in pale gray, blue, and pale green, respectively. The H_β_ atoms of the stable species (Asn, Suc, Asp and *iso*Asp) are shown and colored according to the species color-coding: Asn in blue, Suc in orange, Asp in green and *iso*Asp in red. (B) The kinetic scheme associated with the proposed mechanism with square rectangles embracing the stable species while circles highlight the unstable species (Asn^–^ and Tet^–^). For the sake of clarity, we omitted Gly in the species names.

Interestingly, conflicting results have been reported for the pH dependency of succinimide formation.^2,9^ The acidity of the amide NH group is influenced by the nature of the (*n*+1) amino acid adjacent to Asn, and is expected to affect the reaction kinetics; however, its role has not yet been fully disclosed. It is worth noting that amide NH acidity is a matter of debate, with few experimental data available for N–H p*K*_a_, even for short peptides.^17–20^

Organic textbooks refer to amides as chemical species characterized by a scarce acidity, with a p*K*_a_ between 15 and 20 in the case of secondary amides. For instance, in the case of *N*-methylacetamide (NMA), a p*K*_a_ of 17.7 was estimated in water.^19,21^ Nevertheless, as the complexity of the system increases, determining the p*K*_a_ of secondary amides becomes more challenging, particularly in the context of peptides and proteins.^17,19,22^ However, the occurrence of processes involving the abstraction of amide protons at physiological pH,^23^ showing rates significantly faster in milder alkaline solutions (pH ≈ 8–9),^2,9^ demonstrates that the deprotonation can occur under certain circumstances. The hypothesis is thus that local amino acid environment, the Asn backbone conformation, and nitrogen solvation, collectively exert influence on the acidity of NH groups in proteins, leading to a significantly lower p*K*_a_ for the residue undergoing deamidation compared to that of NMA.

In light of these considerations, we sought to decipher the mechanism of the deamidation reaction in a dipeptide, namely Ace-AsnGly-NH_2_ – hereafter referred to as AsnGly – to be considered as a model of the unstructured regions of proteins. Given the inherent complexity of the mechanism (see Figure 1A), we employed a side-by-side experimental and computational approach that enabled a multiscale, in-depth, and extensive characterization of the reaction. Thus, we combined the modeling of the free energy profile of the deamidation reaction as obtained by a QM/MM approach with Attenuated Total Reflection Infrared (ATR-IR) and Nuclear Magnetic Resonance (NMR) spectroscopies to characterize the comprehensive deamidation reaction kinetics of AsnGly.

## Results and Discussion

### Theoretical-Computational Modeling of the AsnGly Deamidation in Solution

According to the most widely accepted mechanism, the formation of succinimide, which is recognized as the rate-determining step,^8,9,14,23^ requires three steps: deprotonation of the N_*n*+1_ residue, intramolecular cyclization, and final deamination. These steps were therefore incorporated in the kinetics modeling of the reaction.

- In silico estimation of the p*K*_a_

Employing two different theoretical-computational strategies (QM-p*K*_a_/QM-p*K*_acorr_ and PMM-p*K*_a_), described in the Theory and Methods section of the Supporting Information, we found that the acidity of NH amide on the residue following Asn resulted consistently higher (see Figure 2A) than that of NH amide in NMA, a secondary amide commonly used as a model for the protein backbone.^24–26^ This finding, a possible consequence of intramolecular hydrogen bonds stabilizing Asn^–^ species (see Figure 2B), agrees with the well-known sensitivity of p*K*_a_ to neighboring electrostatic variations.^27–29^

**Figure 2:**
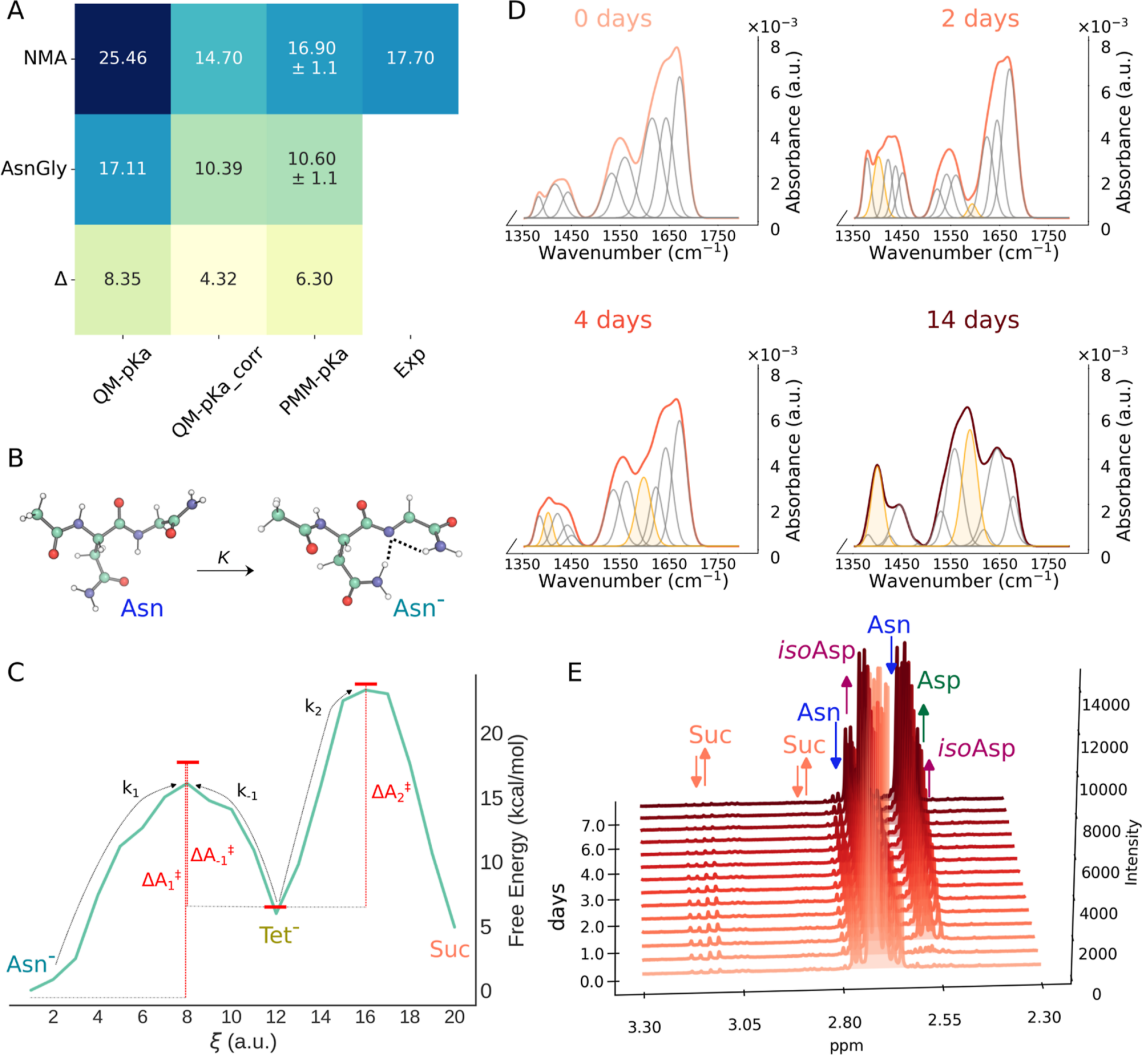
AsnGly dipeptide deamidation mechanism and kinetics explored through theoretical-computational calculations, ATR-IR and NMR spectroscopies. (A) Heatmap of acid dissociation constant (p*K*_*a*_) for NH amides in NMA and AsnGly, estimated via different computational approaches. The error associated with the PMM-p*K*_a_ values reflects the experimental uncertainty in the gas-phase acidity^38^ ( $\Delta G_{AH\overset{A}{\to }+H^{+}}^{gas}$ in Equation S8). QM-p*K*_a_ and QM-p*K*_acorr_ are calculated according to Muckerman et al.^39^ Experimental (Exp) value taken from Sigel and Martin.^19^ (B) 3D structures of neutral and anionic forms of AsnGly. (C) AsnGly Helmholtz free energy profile along the reaction coordinate (ξ) for the two-step succinimide formation. (D) Time evolution of ATR-IR spectra of the AsnGly solution (20 mM) in PB at pH 8. Below each AsnGly ATR-IR spectrum, the corresponding Gaussian deconvolution at that time point is shown as solid gray lines. The Gaussian components assigned to the xAsp vibrational modes are colored and filled in pale orange. (E) Stacked 1D-NMR spectra of the AsnGly solution (20 mM) in PB at pH 8 at different time points expanded in the region of H_β_ hydrogens (2.30–3.30 ppm), framed by square boxes in Figure 1B. The arrows indicate signals increasing or decreasing with time for each stable species, colored according to the color-coding defined in Figure 1.

Previous computational studies^17,18,30^ pointed out a potential conformational dependence of the acidity of amide NH groups. Both these effects, inherently included in our PMM-p*K*_a_ calculations, result in an enhanced NH amide acidity (i.e., p*K*_a_ = 10.6).

- Computing the free energy barriers Asn^–^ ⇌ Tet^–^ → Suc

The Helmholtz free energy profile along the reaction coordinate (ξ) was computed for the succinimide formation in the AsnGly dipeptide (Figure 2C) combining a QM/MM approach – the Perturbed Matrix Method (MD-PMM)^31^– with the free energy perturbation method, detailed in the Theory and Methods section of the Supporting Information. Such a procedure, extensively validated in similar systems,^8,32–35^ guarantees an accurate and physically sound estimation of the free energy profile of a reaction occurring in complex environments, i.e. in solution. The resulting profile confirms a two-step reaction in solution, featuring two comparable free energy barriers, divided by the metastable intermediate Tet^–^. In a previous work by our group,^8^ a similar (free) energy pattern was obtained for the Asn-NHMe model compound, suggesting that the main contribution to the stabilization of the negative charges involved in the reaction arises from the semiclassical perturbation generated by the solvent.

- Theoretical estimation of the kinetic constants

By applying the generalized transition state theory (GTST)^36,37^ on the PMM-derived free energy profiles, we computed the kinetic constants for each step of the succinimide formation (*k*_1_, *k*_–1_, *k*_2_ in eq 1 and Figure 1B). Based on this model, by solving the set of ordinary differential eqs (Equations S10–S13) associated with eq 1, we estimated a deamidation half-life in water ranging from 9.3 to over 400 days within the 9.5 to 11.5 p*K*_a_ range (see Figure S2), defined by the uncertainty associated with the experimental value (see Figure 2A and Methods).

### Liquid-phase ATR-IR Spectroscopy: Estimation of Asn Deamidation Kinetics

To monitor the progress of the deamidation reaction in the AsnGly dipeptide at pH 8 in the presence of buffer and 298 K, we employed ATR-IR spectroscopy, targeting the potential variation in the vibrational modes within the three stable species of the reaction (square box in Figure 1B): Asn, Suc, and xAsp. The normalized ATR-IR absorbance spectra over time (Figure S3) revealed differences in the spectral region ranging from 1350–1800 cm^–1^, linked to characteristic IR absorptions of peptides and proteins,^40,41^ such as Amide III (1200–1450 cm^–1^), Amide II (1500–1600 cm^–1^) and Amide I (1600–1700 cm^–1^).^40,42,43^

An in-depth analysis of the ATR-IR data over time indicates that the xAsp formation could be unambiguously detected from the deconvolution of the spectra into Gaussian bands (Figures 2D and S4). Indeed, two peaks, located at 1400–1402 cm^–1^ and 1574–1579 cm^–1^, associated with the symmetric and antisymmetric ν­(COO^–^) vibrations, assigned to the xAsp side chain, become detectable starting 24 h after the preparation of the sample (orange filled-curve in Figures 2D and S4). Given the unambiguous assignment of these peaks, we estimated the relative concentration of xAsp through IR absorption band analysis (Table S1). In contrast, quantifying the concentrations of the other two species – Asn and Suc – proved to be more challenging. Several factors hindered a reliable assignment of their characteristic vibrational modes: (i) the presence of two primary amide groups in the AsnGly dipeptide (the side chain of Asn and the C-terminal cap), which have comparable flexibility; (ii) potential hydrogen-bond-induced peak shifts for Asn; and (iii) limited data on the specific vibrational features of the backbone-conjugated succinimide intermediate. Consequently, we could not assign specific IR bands to Asn or Suc with high confidence. Although we were unable to quantify the absolute concentration of all the species present simultaneously in the solution, we observed that, with time, the relative xAsp concentration follows an exponential growth, allowing the estimation of a deamidation half-life of 3.5 days (Figure S5), in good agreement with previous experimental data on similar systems.^3,4,10,44^ Therefore, despite the fact that the complete quantification of all the stable species involved in the reaction was not feasible, the distinct appearance of xAsp-related peaks indicates ATR-IR spectroscopy as a valuable qualitative tool for the detection of Asn deamidation.

### NMR Spectroscopy Comprehensive Quantification of the Deamidation Species

To provide a quantitative characterization of the species in the AsnGly solution over time, we turned to NMR spectroscopy, a gold standard method for elucidating the kinetics of chemical reactions.^45^ Two solutions of the AsnGly dipeptide were prepared at pH 8 and at pH 6 and kept at ∼298 K using phosphate buffers (PB; see Methods in Supporting Information). A series of one-dimensional (1D) ^1^H spectra was acquired at various times from solution preparation, in three independent experiments. In PB at pH 6, after three months, nearly one-third of the initial AsnGly was deamidated. Remarkably, at pH 8 after 8 days, nearly 90% of the peptide was deamidated (Figures 2E and S6). These results highlight the remarkable pH dependence of the Asn deamidation reaction.

As the AsnGly deamidation reaction proceeds, its concentration decreases with the appearance of three new compounds: namely, the succinimide intermediate and the final products AspGly and *iso*AspGly. All these species were unambiguously identified by 2D NMR spectroscopy (Figures S7, S8 and S9 and Table S2), according to the available literature data.^46,47^ At pH 8, the expansion of the 1D-NMR spectra in the region of the H_β_ signals at all the analyzed time points (Figure 2E), shows the decrease in the peaks associated with Asn species and the increase in the peaks corresponding to the appearance of the reaction products (Asp and *iso*Asp). After 2.25 days, the intensity of the signals associated with the Suc species reached its maximum value, and from that point, a gradual decrease was observed. Integration of the 1D spectra allowed us to follow specific signals for each compound, thereby enabling the calculation of the relative composition (in moles) of the reaction mixtures at pHs 6 and 8 across various time points during the reaction (Figure S10 and Tables S3 and S4).

### Uncatalyzed Asn Deamidation in Solution

Despite the influence of buffers on deamidation reaction kinetics having been documented over two decades ago,^9,48^ their molecular effect on the reaction mechanism is still unclear. Extensive experimental studies revolving around the role of different catalysts on the deamidation mechanism,^9,48^ suggested that at neutral and basic pH the reaction follows an apparent general-base-catalyzed reaction. Seeking to minimize the bias in integrating NMR-derived equilibrium of species in solution with the theoretical molecular description of the mechanism, we have performed, for the first time, NMR measurements of deamidation kinetics in water starting at pH 8.2 without buffers and monitoring both the pH and the species population changes along the reaction course. In such experimental conditions, the average deamidation half-life time resulted about 8 times longer with respect to the experiment performed in PB, with only a ≈ 20% of the reactant being deamidated after 10 days (see Table S5). By monitoring the variation of pH over time, it has been possible to appreciate how the downshift in pH follows an exponential trend, reaching an estimated minimum value of 7.7 after approximately 40 days (see Figure S11). The reduction of 0.5 unit of pH along the course of the reaction could be alone responsible for reducing the reaction rate by 3 times, according to the proposed deamidation kinetic scheme (see Figure S12).

Therefore, the observed differences in the reaction kinetics when comparing the signals measured in the presence and in the absence of the PB cannot be explained by the pH variation only. However, the known ability of hydrogen phosphate to participate in proton-transfer networks and general acid–base catalysis in biological systems may support the hypothesis that the buffer ion behaves as a silent assistant during the deprotonation step.^49–54^ The hypothesis is that the hydrogen phosphate ion has the ability to increase the acidity of the backbone NH group via hydrogen phosphate-NH hydrogen bonding, proton abstraction, and proton shuttle mechanism. These effects are additive to the buffering effect’s inherent capacity to resist a decrease in pH.

It is worth noting that, given the similar effect observed in several buffers^9^ on the reaction kinetics, the direct involvement of hydrogen phosphate ion in the following steps of the reaction has not been considered.

### NMR-Derived Kinetic Constants

NMR spectroscopy, therefore, allowed the determination of the deamidation half-life times for the model dipeptide AsnGly with and without PB. As discussed in the previous section, in the absence of PB buffer, the reaction occurs on a longer time scale. This could be a consequence of the reduced acidity of the backbone NH amide without the hydrogen phosphate ion acting as a general base catalyst, along with the decrease of hydroxide ion concentration in solution over time, as demonstrated by the pH drop observed in this work (see Figure S11) and in previous experiments.^2^ Under this hypothesis, the deamidation reaction mechanism would not be affected by the presence of the buffer.

In summary, the effect of the buffer is considered to accelerate the reaction kinetics by influencing the equilibrium constant of the deprotonation step (*K*), whereas the overall mechanism of the reaction (see the Scheme in Figure 1) is expected to be preserved. To gain further insight into this aspect and shed light on the contribution of each step within the integrated computational and experimental framework proposed, we estimated the step-specific kinetic constants (*k*_1_, k_–1_, *k*_2_, and *k*_3_ as well as the ratio between the Asn and Asn^–^ concentrations, K in eq 1, expressed as p*K*_a_ via Eq S17) by using the concentrations of the species involved as determined by ^1^H NMR spectroscopy. The strategy employed, described in the Supporting Information and schematically illustrated in Figure 3A, involves solving the differential equations of the kinetic model (Equations S10–S13) using as starting guesses the constants obtained by the computational-theoretical procedure, followed by an optimization step to minimize the discrepancy between the predicted and measured concentrations. When analyzing the relative concentration of the stable species in solution without buffer (NB), as determined by three independent NMR experiments, we could utilize the PMM-derived kinetic constants and p*K*_a_ as initial guesses of the procedure, which resulted in a good agreement between the simulated and the experimental variation of the concentration of the species over time (see Figure S13). Furthermore, the experimentally derived constants are in good agreement with the theoretical kinetic data (Table 1 and S6), validating the deamidation kinetic model and the mechanism proposed in this work. On the other hand, the p*K*_a_ value estimated in water by PMM might not be a valid initial guess for analyzing the deamidation kinetics in PB, given the hypothesized role of the buffer in acidifying the NH of the amide. Thus, the procedure depicted in Figure 3A was repeated iteratively using a range of p*K*_a_ initial values ranging from the theoretical estimate (10.6) to 9 (see Table S7). Such a strategy avoids the need to rely on a theoretical estimate of the buffering effect on the p*K*_a_, which would require further modeling of the entire dipeptide-phosphate complex, a task that falls outside the scope of this study. Indeed, the kinetic constants (reported in Table 1), obtained by averaging the kinetic constants that resulted in the lowest average RMSE at a fixed p*K*_a_ guess (9.3 and 9.4, see Table S7), support the hypothesis of a buffer-independent deamidation mechanism, as demonstrated by the high similarity of the Suc formation kinetic constants between the two experimental conditions as well as with the theoretical constants (i.e., the cyclization/deamidation steps are buffer-independent, but the depronotation step is subject to general base catalysis). Interestingly, a similar increase in the rate constant was observed by Capasso and co-workers^9^ when the deamidation occurs in the presence of phosphate buffers (eq 1 and Table 1 in that paper).

**Figure 3:**
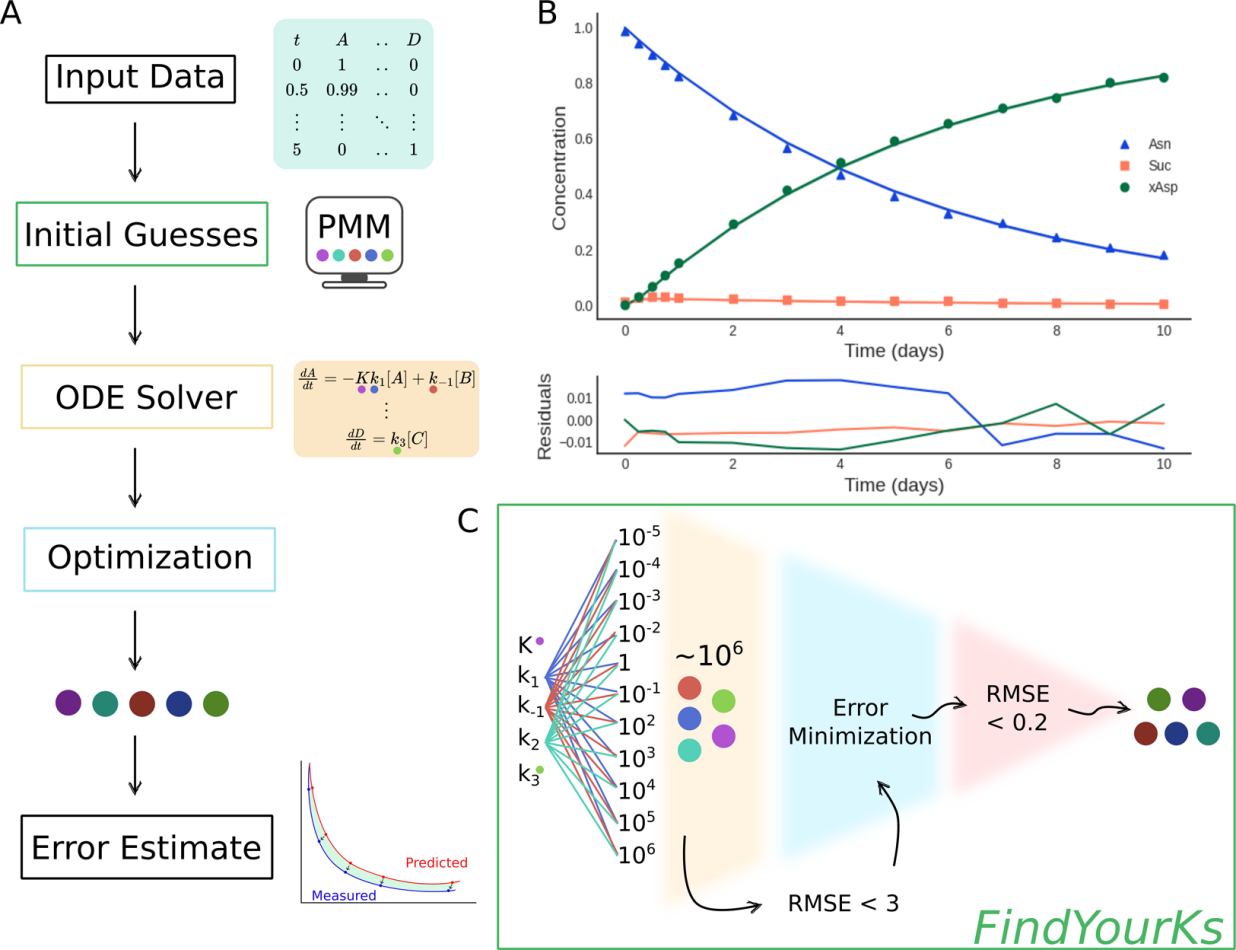
Single-step kinetic constants estimation by the fitting of NMR relative concentrations of species (solid points in panel (B) in PB at pH 8 and 298 K. (A) Overview of the fitting and optimization workflow utilized to extract the kinetic constants from the experimental data, starting from the computationally determined constants. (B) Theoretical kinetic constants utilized as the initial guesses (see Methods) resulted in a satisfactory representation of the NMR concentration trend in three independent experiments (see Figure S7) in PB at pH 8, with an RMSE less than 0.017. The average simulated variation of relative populations is shown as solid lines. The differences between the experimental data and the numerical simulation of the concentration changes (residuals) for each species are reported in the bottom panel, using the same color scheme as in the main graph. (C) Schematic illustration of the strategy developed (FindYourKs - see Supporting Information) for the estimation of the kinetic constants by iteratively solving eqs S10–S13 and through the error minimization between the predicted and the experimental values (see Methods in Supporting Information for further details).

**Table 1:** Comparison Among the Deamidation Kinetics Constants (s^–1^) Experimentally Determined with PB at pH 8 (NMR and ATR-IR) and without Buffer (NB) at pH 8 (NMR) with those Calculated by the MD-PMM Procedure

	calc	NMR NB	NMR PB pH 8	ATR-IR pH 8
p*K*_a_	10.6 ± 1.1	10.3 ± 0.1	9.1 ± 0.06	9.1
*k*_1_	3.7 · 10	2.5 · 10 ± 12.6	3.9 · 10 ± 3.3	4.0 · 10
k_–1_	9.2 · 10^6^	5.2 · 10^6^ ± 1.13 · 10^6^	7.1 · 10^6^ ± 4.3 · 10^4^	8.4 · 10^6^
*k*_2_	6.7	6.9 ± 0.06	7.2 ± 0.23	9.1
*k*_3_	-	2.6 · 10^–5^ ± 3.7 · 10^–6^	8.2 · 10^–5^ ± 5.4 · 10^–7^	5.2 · 10^–5^

This analysis was then extended to NMR-derived ATR-IR spectroscopy data relative concentrations of species (Table S8 and Figure S15; see Methods in Supporting Information for further details), which provided similar constants (Table 1), further corroborating the hypothesized deamidation mechanism. In addition, analogous outcomes were also attained for a tetrapeptide, using the NMR data by Pilhal and colleagues^15^ (see Figure S16). To further test whether the adopted simplification of succinimide hydrolysis (i.e., treating succinimide hydrolysis as a single irreversible step forming xAsp, rather than a reversible Suc to Asp, *iso*Asp equilibrium) could have had an impact on both the succinimide formation mechanism and on the extrapolated kinetic constants, our kinetic model was extended to include the equilibrium between Suc, Asp and *iso*Asp (see Figure S17). The close agreement between the kinetic constants obtained with the extended model and those derived from the simplified scheme confirmed that the adopted approximation was legitimate and that the succinimide hydrolysis equilibrium does not significantly affect succinimide formation. Finally, to reduce the bias of extracting experimental constants using the computationally determined constants as initial guesses, a strategy was developed (FindYourKs available as a Jupyter notebook, see Supporting Information) to fit the experimental data to the complex kinetic models without any a priori knowledge of the constants, illustrated in Figure 3C (see Supporting Information).

Given the complexity of the model and the high number of variables in the equations, it should be emphasized that the resulting values are numerical solutions that need to be filtered for their physical meaning. The procedure was applied to fit the NB experimental data points, and repeated iteratively using a fixed p*K*_a_ value, comprised between 10.6 and 9.5. Given the high statistical uncertainty associated with the analysis, where random values are assigned to kinetic constants and combined, the results of this analysis should be interpreted in a qualitative-mechanistic perspective. The free energy barriers associated, via the Eyring relation, with the pool of physically relevant solutions of the differential equation system to fit the variation of the concentrations of the NMR-derived species over time are rescaled by the guessed p*K*_a_ value (see Table S9). However, by analyzing the ratio between the free energy barriers of the succinimide formation step, the preserved ratio between the two forward reactions (Asn^–^ → Tet^–^ and Tet^–^ → Suc) could be appreciated. This evidence matches well with the corresponding MD-PMM free energy barriers (see Table 2 and the red lines in Figure 2C), thus reinforcing the proposed two-step mechanism for succinimide formation. Furthermore, it also strengthens our previous hypothesis^8^ on the crucial role of the metastable tetrahedral intermediate (Tet^–^) stabilization.

**Table 2:** Ratio of Free Energies Barriers for the Two-step Succinimide Formation Associated with the Kinetic Constants Obtained at the End of the Filtering Process Imposing a Fixed pKa Value in the Range 10.6 to 9.5^a^

	$\frac{\Delta A_{1}^{‡}}{\Delta A_{-1}^{‡}}$	$\frac{\Delta A_{1}^{‡}}{\Delta A_{2}^{‡}}$	$\frac{\Delta A_{2}^{‡}}{\Delta A_{-1}^{‡}}$
model p*K*_a_ 9.5	1.6	1.0	1.6
model p*K*_a_ 10	1.3	1.1	1.2
model p*K*_a_ 10.3	1.5	1.0	1.4
model p*K*_a_ 10.6	1.4	1.0	1.4
MD-PMM	1.6	0.9	1.8

a The Corresponding MD-PMM Free Energy Barriers are Reported for Comparison. The Error on these Values is Less Than 0.1%. The Average Free Energy Value for Each pKa are Reported in Table S9

## Conclusions

A comprehensive investigation on the deamidation kinetics of the model dipeptide AsnGly has been conducted through the combined use of theoretical-computational methods and spectroscopic (NMR and ATR-IR) techniques. The molecular mechanism and kinetics of succinimide formation were independently assessed through MD-PMM and ^1^H NMR spectroscopy, which yielded convergent results in both the overall kinetics and step-specific kinetic constants. It is noteworthy that the single-step kinetic constants derived from the experiments, with and without buffer, to the best of our knowledge, have heretofore never been disclosed. Furthermore, a numerical procedure was adopted as a blind assay attempting to remove any potential bias, which finally serves as a further validation of the proposed molecular mechanism. The combined experimental and theoretical characterization at the molecular level of the deamidation kinetics revealed the reaction sensitivity to pH, and unambiguously demonstrates the two-step mechanism for the succinimide formation, which was previously only hypothesized.^9^ In summary, unveiling the kinetics and mechanism of this reaction represents a valuable advancement in the comprehensive understanding of deamidation in proteins, opening the way to next-generation algorithms that are crucial in the field of biotherapeutics.

## Supplementary Material



## Notes

Details on the MD-PMM and GTST theoretical foundation, computational and experimental methods, additional characterization data (NMR and ATR-IR), NMR peak assignment for each stable species, raw data of species concentration quantified experimentally, fitting of further experimental data (PDF)

## ∥

Biologics Research Center, Novartis Campus, 4056 Basel, Switzerland
