# Supplementary material for: A Combined Experimental-Theoretical Approach Unveils the Comprehensive Asn Deamidation Kinetics
Source: J Am Chem Soc. 2026 Jul 28;148(31):33727–34. doi: 10.1021/jacs.6c11010 (PMC13474922; doi:10.1021/jacs.6c11010)
Supplement: Supplementary file 1 [file ja6c11010_si_001.pdf]

Supporting Informations for: A combined experimental-theoretical approach unveils the comprehensive Asn deamidation kinetics

Maria Laura De Sciscio,† Rosanna Mosetti,‡ Annalisa D'Arco,‡ Stefano Lupi,‡ Mariana Gallo,¶ Thelma A. Pertinhez,¶ Fabrizio Vetica,† Mauro Giustini,† and Marco D'Abramo*,†

†*Department of Chemistry, University of Rome, Sapienza, P.le A. Moro 5, 00185 Rome, Italy*

‡*Physics Department, University of Rome Sapienza, P.le Aldo Moro 5, 00185 Rome, Italy*

¶*Laboratory of Biochemistry and Metabolomics, Department of Medicine and Surgery, University of Parma, 43125 Parma, Italy*

E-mail: marco.dabramo@uniroma1.it

Theory

The Perturbed Matrix Method

Estimating the electronic properties of complex molecules in aqueous environments requires treating both classic and quantum degrees of freedom. An accurate yet efficient computational approach comprises the application of different levels of theory within the same system. The complex system is divided into a small group of atoms, which are directly involved in the electronic process of interest, and the complementary part of the system, i.e.

the environment. The former, called Quantum Center (QC), is accurately described using Quantum Mechanics (QM) calculations, while the rest of the system can be treated using Molecular Mechanics (MM).

In the Perturbed Matrix Method (PMM), the electronic properties of the QC ($\hat{H}$) are obtained by adding a perturbation operator ($\widehat{V}$), which describes the semi-classic, electrostatic interaction between the QC and the environment, to the gas-phase unperturbed QC electronic properties ($\widehat{H}^0$). The interaction can be recalculated for each MD frame "a posteriori", as shown in Equation S1. This unique feature allows the use of accurate QM level of theory and long timescale MD simulations (MD-PMM), making it a suitable approach for studying chemical reactions of in complex systems.[1–6]

$$\widehat{H} = \widehat{H}^0 + \widehat{V} \tag{S1}$$

The diagonalization of the electronic Hamiltonian matrix of the QC within the environment, at each MD frame, produces a trajectory of eigenvalues and eigenvectors that correspond to the QC perturbed eigenstates and energies. In this work, the MD-PMM calculations – under the atom-based expansion for the diagonal and the dipole-based approximation for the off-diagonal elements of the electronic Hamiltonian matrix[7,8] – were performed to estimate the free energy barriers of the two-step succinimide formation and for the determination of the acidic constant of the N-H group of Gly in the Ace-AsnGly-NH_2 (AsnGly) system, which are described in the next sections.

Estimation of the Laundau Free Energy and the Generalized Transition State Theory

In the context of chemical reactions, the definition of reaction coordinate (ξ) is a conventionally utilized concept. Within the MD-PMM scheme, by performing the calculations along ξ, the free energy variation at specific ξ values ($\Delta A(\xi)$) can be obtained. For each point i

along ξ (solid pink points in Figure S1), the free energy variation between i and the following point $i+1$ is:

$$\Delta A_{i \rightarrow i+1}(\xi) \simeq -k_B T \langle e^{-\beta \Delta \mathcal{U}} \rangle_i \tag{S2}$$

where $\Delta\mathcal{U}$ is the perturbed energy change ($\mathcal{U}_{i+1} - \mathcal{U}_i$), which can be estimated by diagonalizing the perturbed electronic Hamiltonian matrix, as obtained by the PMM calculation. In the equation, $\beta = 1/k_B T$, where k_B is the Boltzmann constant and T is the thermodynamic temperature.

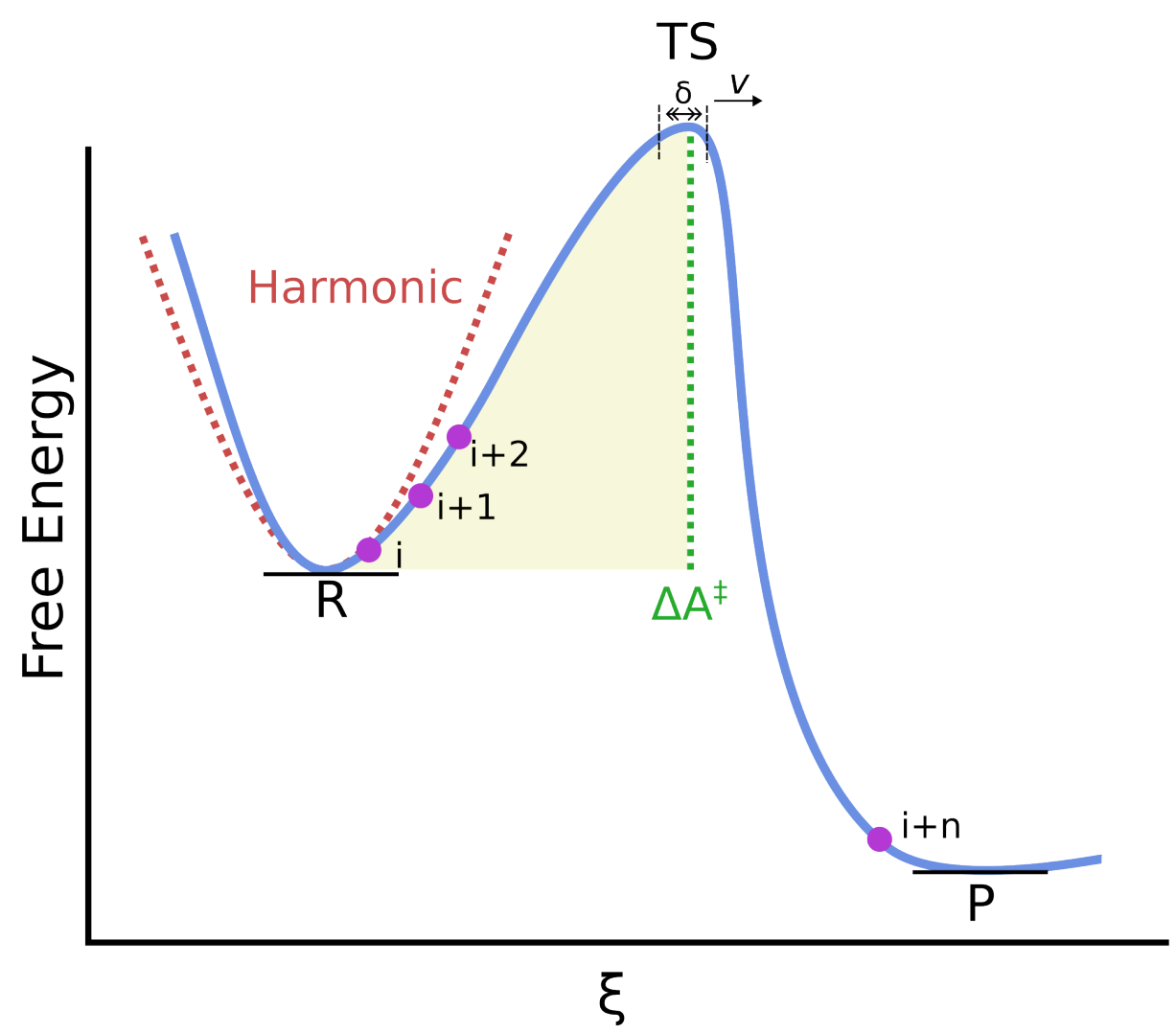

Figure S1: Pictorial representation of the free energy variation along the reaction coordinate (ξ) for a chemical reaction in solution. The yellow area is a graphical representation of $\int_{\xi_{Min}}^{\xi_{Max}-\delta/2} e^{-\beta \Delta A(\xi)} d\xi$ in Equation S3.

.

As previously described,[1,2,4,6] the estimation of $\Delta A(\xi)$ allowed us to apply the Generalized Transition State Theory (GTST), a theory that has been proven to be efficacious in describing the kinetics of chemical reactions in solution, as in the case of this work. The GTST indeed permits the inclusion of the complex nature of the reaction coordinate, by explicitly computing the velocity (v) with which the Transition State (TS) transits through the tiny TS region (δ), and takes into account the deviation from the harmonicity in the reactant (R) and TS regions by analyzing the shape of the free energy profile along ξ (yellow

area in Figure S1). Therefore, according to the GTST, the kinetic constant for a general reaction $R \rightarrow TS \rightarrow P$ going through a unique TS state, can be expressed as:

$$k = \frac{e^{-\beta \Delta A^{\ddagger}}}{\int_{\xi_{Min}}^{\xi_{Max}-\delta/2} e^{-\beta \Delta A(\xi)} d\xi} \frac{v}{2} \tag{S3}$$

where the integral limits (ξ_{Min}, $\xi_{Max} - \delta/2$) define the reactant ensemble ξ area, from the energy minimum, i.e. the reactant, to the TS immediately preceding point. $\Delta A(\xi)$ is the Landau free energy variation along ξ, while $\Delta A^{\ddagger}$ is the free energy variation between the transition state and the reactant (Figure S1). When the shape of the reactant area is fully quadratic (dotted red line in Figure S1) and the TS traversing velocity (v) is approximated to a normal mode fluctuation, Eq. S3 becomes the well-known Eyring equation:

$$k_E = \kappa \frac{k_B T}{h} e^{-\Delta A^{\ddagger}/RT} \tag{S4}$$

where R is the ideal gas constant, and κ the transmission coefficient. For a thoughtful explanation of the theoretical foundation beyond the concepts here discussed, please refer to the following references: Amadei et al.[9], Amadei et al.[10],Peters[11], Nardi et al.[1].

pKa Theoretical Determination

Given the relevance of an accurate estimate of the pKa of the Gly NH in comprehending the deamidation reaction in solution, we employed a previously developed PMM-based framework[12] to rigorously estimate the acidity of the amide NH group in AsnGly.

The deprotonation reaction of AsnGly reactant species, referred to as Asn (Figure 1 A), into its conjugate base Asn$^-$, can be expressed as:

$$Asn \rightleftharpoons Asn^- + H^+ \tag{S5}$$

The pKa of the Asn species can be expressed as

$$pKa = -log\frac{[Asn^-][H^+]}{[Asn]} = \frac{\mu^\circ_{Asn^-} + \mu^\circ_{H^+} - \mu^\circ_{Asn}}{2.303k_BT} \quad \text{(S6)}$$

For diluted species, as in the case of this work, the activity coefficient is assumed unitary and, therefore, μ° corresponds to the standard chemical potential. The standard chemical potential variation occurring upon deprotonation ($\Delta\mu_{Asn\rightarrow Asn^-} = \mu^\circ_{Asn^-} - \mu^\circ_{Asn}$) coincides, at constant volume, with the Helmholtz free energy variation (ΔA) plus the vibrational contribution to the deprotonation energy (ΔA^{sol}_{vib}), i.e. $\Delta\mu_{Asn\rightarrow Asn^-} \backsimeq \Delta A + \Delta A^{sol}_{vib}$.
The standard chemical potential of the solvated proton ($\mu^\circ_{H^+}$), reported in Equation S7, is obtained from the experimental solvation energy of the proton ($\Delta\mu_{H^+}$), the gas-phase standard free energy variation of the deprotonation reaction ($\Delta G^{gas}_{AH\rightarrow A+H^+}$) and the QC unperturbed ground state energy change upon deprotonation, ΔU^0_{el}.

$$\mu^\circ_{H^+} = \Delta\mu_{H^+} + \Delta G^{gas}_{Asn\rightarrow Asn^-+H^+} + k_BT\ln(k_BT\frac{\rho^\circ}{P^\circ}) - \Delta U^0_{el} - \Delta A^{gas}_{vib} \quad \text{(S7)}$$

where the term $k_BT\ln(k_BT\rho^\circ/P^\circ)$ is the correction factor for the gas to solution state change, with ρ° being the solution standard molecular density (1 M) and P° the gas phase standard pressure (0.1 MPa).
Finally, using Equations S6 and S7 and assuming $\Delta A^{sol}_{vib} \cong \Delta A^{gas}_{vib}$, the pKa expression becomes:

$$pKa = \frac{\Delta\mu_{H^+} + \Delta G^{gas}_{AH\rightarrow A+H^+} + k_BT\ln(k_BT\frac{\rho^\circ}{P^\circ}) + \Delta A - \Delta U^0_{el}}{2.303RT} \quad \text{(S8)}$$

The Helmholtz free energy change upon deprotonation can be efficiently obtained through MD-PMM calculations. In this work, the atom-based expansion approximation was utilized to estimate the Helmholtz free energy change. Following the derivations reported in Zanetti-Polzi et al.[12], D'Annibale et al.[13], the ΔA can be expressed as:

$$\Delta A \cong \frac{k_BT}{2}\ln\frac{\langle e^{\beta\Delta\mathcal{U}}\rangle_{Asn^-}}{\langle e^{-\beta\Delta\mathcal{U}}\rangle_{Asn}} \quad \text{(S9)}$$

Materials and Methods

Computational Strategy

The estimation of Ace-AsnGly-NH_2 (AsnGly) deamidation kinetics required the calculation of i) NH acidity to estimate the pKa; ii) the Helmholtz free energy of activation for the ring-closure step; iii) the Helmholtz free energy of activation for the deamination step (see Figure 1A). Specifically, for points ii) and iii) it was extended the methodological framework already used in a previous work[4] on the succinimide formation on the model compound AsnNHMe. The reactions were modeled using the Molecular Dynamics-Perturbed Matrix Method (MD-PMM) scheme, a theoretical-computational hybrid quantum mechanics/molecular mechanics (QM/MM) approach featuring remarkable accuracy at a reasonable computational cost (see previous section). For each reaction step, the kinetic constants were estimated following the Generalized Transition State Theory (GTST), discussed in the Theory section. Two methodologies were applied to the calculation of the acid dissociation constant. One is based on the theoretical derivation discussed in the previous section, which involves the MD-PMM scheme. In previous works,[12,13] the employed methodology demonstrated its ability to capture acidity variation of ionizable side chain groups in proteins. In this work, to model this thermodynamic property, we used as QC a model compound for the backbone amide,[14] namely the N-methylacetamide (NMA). The procedure required calculating the ground state electronic properties of NMA in neutral (NH) and anionic (N^-) states, as well as one MD simulation of the peptide in water for each protonation state. Given the complexity of treating the QM/MM boundary region (i.e. the link atoms) in the protein backbone, we also applied a full-QM methodology, developed by Muckerman et al.[15]. According to such a method, on the optimized structure of interest, a single point calculation using the Conductor-like Polarizable Continuum solvation Model (CPCM) to model the implicit solvent and the solvation, along with vibrational frequencies calculations, is required for each state (neutral and anionic). In this case, the whole AsnGly was used for the calculations.

Quantum Chemical Calculations

All the Quantum Chemical calculations were performed using Gaussian16.[16] The initial coordinates of the optimized structure of the trans-NMA in the anionic form were retrieved from the work of Kelemen and coworkers.[17] The neutral form of NMA was generated by adding a hydrogen ion to the anionic nitrogen atom using the Avogadro software,[18] leaving the nuclear position of the other atoms unchanged. For each protonation state, the structures were optimized at the CCSD/Def2TZVP level, and the frequencies checked to verify that was a minimum. Then, these structures were subjected to another optimization step at the same level of theory but using a more extensive basis set, specifically aug-cc-pVDZ. Once verified that the stationary point was a minimum, the ground state electronic properties (energies, dipoles, and ESP charges) at the CCSD(T)/aug-cc-pVDZ level were utilized in the MD-PMM scheme ($\widehat{H}^0$). For the QM-pKa procedure,[15] the calculations were performed on the whole AsnGly in the neutral and anionic NH amide form of Gly. Due to the high number of atoms in the system, calculations using CCSD was not feasible as the level of theory resulted in a prohibitive computational cost. Consequently, the calculations were performed using B3LYP and 6-311++G** as the basis set. The initial coordinates of the neutral dipeptide were obtained by extracting the most sampled conformation among a subset of conformations having a distance N-C_γ minor than 4 Å from an MD simulation of the dipeptide in water. The corresponding anionic form was generated by removing the hydrogen ion from the NH amide of Gly. The structures were optimized at the B3LYP/6-311++G** and their nature was verified by calculating the vibrational frequencies of the stationary point found. To enable a proper comparison between NMA and AsnGly pKa, using the QM-pKa procedure, the same calculations were also conducted on NMA, utilizing the same level of theory and basis set of AsnGly.

Molecular Dynamics Simulations

Neutral and anionic NMA as well as neutral and anionic AsnGly were simulated for 150ns without constraints in the NVT ensemble at 300 K. Conversely, the MD simulations of the dipeptide required for estimating the free energy variation along the reaction coordinate for the two-step succinimide formation, in the MD-PMM context, were performed constraining the reaction coordinate. This procedure ensured the calculation of the Landau free energy and thus the application of the GTST. These simulations were performed in the NVT ensemble, at 300K, and lasted 150 ns. The force field used for all the simulations of the dipeptide was Amber99sb-ildn. Tet^-, Suc, and NMA parameters were obtained using ACPYPE.[19] Asn^- parameters were manually set equal to the of Asn with the exception of all the bonded parameters involving the missing hydrogen ion, which were removed. The parameters for the anionic form of NMA were obtained using the same strategy described above for Asn and Asn^-.

All the simulations were performed with Gromacs software package.[20] The solute molecule was centered in a cubic box and subsequently filled with water molecules, using the TIP3P model. Na^+ and Cl^- ions were added to the simulation box to ensure electroneutrality (the final NaCl concentration was 0.15 M). Following a minimization step using the steepest descent algorithm, a series of consecutive NVT equilibration steps were performed by adjusting the simulation box size to reproduce the experimental density of the water, as previously described.[21,22] The velocity-rescale algorithm[23] was applied to control the temperature of the simulations. Long-range electrostatic interactions were estimated according to the Particle Mesh Ewald (PME) scheme,[24,25] with a cut-off of 1.1 nm. The same cut-off was utilized for the non-electrostatic long-range interactions, using the Verlet algorithm to generate the neighbor list.[26]

MD-PMM calculations

The MD-PMM procedure was employed to estimate the free energy profile along the reaction coordinate for the two-step succinimide formation and for the NH amide pKa. In this work, the QC (AsnNHMe) electronic properties were taken from our previous work.[4] The perturbation generated by all the remaining atoms ($\widehat{V}$ in Equation S1) was estimated using the MD simulations of the dipeptide in water. Employing the theoretical-computational framework discussed in the Theory section, we estimated the pKa of the NH amide of NMA and the dipeptide, using in both cases NMA as QC, via Equation S8. In that equation, the experimental value[27] for the free energy variation upon deprotonation in gas phase $\Delta G^{gas}_{NMA \rightarrow NMA^{-}+H^{+}}$ was used, as previously reported.[12,13] The chemical potential variation due to the proton solvation ($\Delta\mu_{H^{+}}$) was also retrieved from previous experiments.[28]

Chemicals

The dipeptide AsnGly, N-terminal acetylated and C-terminal amidated, was purchased as lyophilized powder from GenScript (UK). Phosphate buffer (PB) was prepared by titrating with concentrated HCl a solution of Na_2HPO_4 prepared by dissolving the salt (Sigma-Aldrich) in the suitable amount of ultrapure water (18.2 MΩ/cm) from Arium pro UV system (Sartorius Stedim Biotech). Once the desired pH (6 or 8) was reached, the buffer solution was brought to volume in a volumetric flask. pH was measured using a glass combined electrode (Hamilton MiniTrode) connected to a digital pH-meter (Hanna pH301 pH/ion-meter). The solution preparation was performed at room temperature by weighing the appropriate amount of AsnGly, typically 2-4 mg, into an Eppendorf vial and then adding the PB buffer at the required pH. Samples intended for NMR measurements were prepared in a 5% (by volume) mixture of D_2O/H_2O. In case of NB experiments, the same amount of AsnGly was dissolved in water and a solution of NaOH was added until a pH of 8.2 was reached. Once prepared, the solutions were kept at room temperature ($\sim$ 298 K).

Attenuated-Total-Reflection Infrared Spectroscopy (ATR-IR): experimental set-up and data collection parameters

Attenuated-Total-Reflection Infrared (ATR-IR) absorption spectra were collected using a Bruker Vertex 70v Michelson spectrometer (Bruker Corp., Billerica, MA, USA) equipped with a monolithic ATR–Diamond single reflection module and a DLaTGS wide range detector. The spectroscopic measurements were carried out at room temperature and under vacuum conditions to minimize interferences induced by water vapor and CO_2 absorptions. The buffer spectrum was collected immediately prior to each sample measurement, using the same experimental settings as the AsnGly solution. For each ATR-IR measurement, 5 μl of the solution (dipeptide solution and/or buffer) were directly dropped onto the ATR diamond crystal, collecting several repetitions of 128 scans between 50–6000 cm^{-1} with a spectral resolution of 4 cm^{-1} and then the ATR crystal was cleaned with ethanol (purity $>$ 90 %), distilled water and lens tissue, to eliminate spurious signals. The AsnGly solution at pH 8 was investigated daily for two weeks at exactly the same time each day to monitor the Asn deamidation kinetics. ATR-IR data pre- and post-processing (such as absorbance calculation, baseline correction, water subtraction, ATR advanced correction, cut and average, and Gaussian fitting) were performed using OPUS 8.2 software (Bruker Optics) and dedicated algorithms for weighted fittings based on MATLAB (ver. 2020, MathWorks Inc., Natick, MA, USA).[29–32] Dipeptide analysis was focused on the 1350-1800 cm^{-1} spectral region associated with the amide I, II and III absorption bands.[33–35] Each spectrum was normalized to the area under the spectrum in the 1350-1800 cm^{-1} interval and deconvoluted into single spectral components. The minima of 2nd-derivative absorption spectrum were used to obtain the spectral frequencies as starting points for multiple Gaussian fitting,[36,37] performed with OPUS 8.2 software considering the residual error (RMSE) value as goodness-of-fit parameter.[30,31,33,36] The characteristic frequencies were assigned to specific vibration modes,[33,35] including the absorption bands attributable to xAsp. The integrated areas of their convoluted bands were considered.[33,35] The obtained values for the xAsp areas were

normalized to the maximum value of the xAsp area, which was obtained on day 11 of the analysis.

NMR measurements

All NMR measurements were performed at 298 K using either a Bruker Avance 400 spectrometer (Bruker Corp., Billerica, MA, USA) equipped with a 5 mm Prodigy cryoprobe or a JEOL 600 MHz ECZ600R spectrometer (JEOL Inc., Tokyo, Japan) equipped with a 5 mm ROYAL probe. A series of one-dimensional (1D) ^{1}H spectra were collected at different time points (see Tables S1 and S2) from their preparation (t0) up to nearly three months in the case of samples at pH 6.0 and up to 10 days for PB pH 8 and NB. For the ^{1}H-1D experiments, a pulse sequence with excitation sculpting and gradients for water suppression (the *zgesp* pulse program) was used with the following instrumental parameters: spectral width, 6400 Hz; recycle delay, 1 to 5 s; 8 dummy scans; 32 transient scans; 65-K time domain data points. For chemical shift assignment and identification of the different reaction species, the following two-dimensional (2D) spectra were recorded in the Bruker Avance 400 spectrometer: ^{1}H-^{1}H TOCSY, ^{1}H-^{1}HROESY, ^{1}H-^{13}C HSQC, and ^{1}H-^{13}C HMBC. The ^{1}H-^{13}C HSQC spectra used an HSQC sequence with Echo/Antiecho-TPPI (Time Proportional Phase Incrementation) acquisition mode in F1, sensitivity improvement, and gradient and shaped (selective) pulses (the *hsqcetgpsisp2.2* pulse program). The spectra had a spectral width of 4802 Hz and 4528 Hz for the ^{1}H and ^{13}C dimensions, respectively, 80 or 128 t1 increments, each with 2048 complex data points, 8 transients, and a relaxation delay of 1.0 s. The ^{1}H-^{1}H TOCSY spectra (80 ms of mixing time) used a homonuclear Hartman-Hahn transfer using MLEV17 sequence for mixing, two power levels for excitation and spinlock, and water suppression was achieved using a 3-9-19 pulse sequence with gradients (the *mlevgpph19* pulse program). The spectra had a spectral width of 3605 Hz in both the direct and indirect dimensions, 200 t1 increments, each with 2048 complex data points, 16 dummy scans, 2 transients, and a relaxation delay of 2.0 s. The ^{1}H-^{1}H ROESY spectra (250 ms of mixing

time) used a sequence with cw spinlock for mixing, while water suppression was achieved using a 3-9-19 pulse sequence with gradients (the *roesygpph19* pulse program). The spectra had a spectral width of 3605 Hz in the direct and indirect dimensions, 160 t1 increments, each with 2048 complex data points, 16 dummy scans, 8 transients, and a relaxation delay of 1.0 s. NMR data acquisition, processing, and analyses was performed using the Bruker software package TopSpin.

The 600 MHz JEOL spectrometer was used to record the 1D-^{1}H and 2D ^{1}H-^{13}C HSQC and ^{1}H-^{13}C HMBC spectra of the three samples at t0, and at 90 days for the sample at pH 6. The 1D spectrum used watergate and dante presaturation for solvent suppression. The spectral width was 9000 Hz; 3 s, the recycle delay; 4 dummy scans and 64 transients scans were recorded with 2088 time domain data points. For the ^{1}H-^{13}C HMBC, a hmbc sequence with wet and dante presaturation for solvent suppression was used. The spectra had spectral widths of 9002 Hz and 27162 Hz for the ^{1}H and ^{13}C dimensions, 256 t1 increments, each with 2048 complex data points, 80 transients, and a relaxation delay of 1.0 s. The ^{1}H-^{13}C HSQC, used watergate and dante presaturation schemes for solvent suppression. The spectra had spectral widths of 6600 Hz and 6795 Hz for the ^{1}H and ^{13}C dimensions, 128 t1 increments, each with 2048 complex data points, 4 dummy scans, 20 transients, and a relaxation delay of 1.0 s. NMR data acquisition and processing were performed using the JEOL software package Delta v6.1 and analysed using MestReNova v14.3.3 (Mestrelab Research S.L.) software.

Analysis of experiment-derived species concentrations

The measure of the relative concentrations of each of the stable species (Asn, Suc, Asp, and *iso*Asp) simultaneously present in the AsnGly solution during the deamidation reaction allowed the investigation of the reaction mechanism by comparing the values of the kinetic constants determined theoretically and experimentally. The main focus of this work was the determination of the kinetic constants of all the processes occurring during the transformation of Asparagine into the Succinimidic intermediate, which is generally accepted - and

confirmed by this work - as the rate-determining step of the deamidation reaction.[38–41] For this purpose, the amount of Asp and *iso*Asp was summed and treated as a single product, termed xAsp. Based on our knowledge of the overall reaction, we built a set of ordinary differential equations describing the transformation of Asn to xAsp, including an initial deprotonation stage and the formation of the metastable tetrahedral intermediate (Tet$^-$; see Scheme 1), reported in Equations S10 - S13.

$$\frac{d[Asn^-]}{dt} = -k_1[Asn^-] + k_{-1}[Tet^-] \tag{S10}$$

$$\frac{d[Tet^-]}{dt} = k_1[Asn^-] - (k_2 + k_{-1})[Tet^-] \tag{S11}$$

$$\frac{d[Suc]}{dt} = k_2[Tet^-] - k_3[Suc] \tag{S12}$$

$$\frac{d[xAsp]}{dt} = k_3[Suc] \tag{S13}$$

where the concentration of the deprotonated reactant Asn^- is defined on the pH-dependent pre-equilibrium between the protonated and deprotonated reactant (Asn and Asn^-), i.e. $[Asn^-] = K[Asn]$. When extending the deamidation kinetic scheme to comprise the complete, non-approximate, hydrolysis of succinimide intermediate into Asp and *iso*Asp, the differential equations describing the variation of Suc, Asp and *iso*Asp species concentration over time become:

$$\frac{d[Suc]}{dt} = k_2[Tet^-] - k_4[Suc] - k_5[Suc] + k_{-4}[Asp] + k_{-5}[isoAsp] \tag{S14}$$

$$\frac{d[Asp]}{dt} = k_4[Suc] - k_{-4}[Asp] \tag{S15}$$

$$\frac{d[isoAsp]}{dt} = k_5[Suc] - k_{-5}[isoAsp] \tag{S16}$$

Such equations were first resolved using the kinetic constants calculated by means of MD-PMM as an initial guess. The initial guess for the succinimide hydrolysis kinetic constant (k_3), which was not estimated via the MD-PMM approach, was set to 10^{-4} and 10^{-6} for pH 8 and 6, respectively, according to previous NMR experiments.[42] Note that the ratio between the Asn and Asn^- concentrations, expressed by the quantity K (K = $log_{10}\left(\frac{[Asn^-]}{[Asn]}\right)$), can be easily obtained as a function of pH by rearranging the Henderson-Hasselbalch equation:

$$K = 10^{(pH - pKa)} \tag{S17}$$

To solve the above equations, the PMM-pKa value was utilised and the pH value was set equal to the one used in the NMR experiments (pH 6 and 8). The obtained kinetic constants were further optimized by minimizing the differences between the predicted concentrations and the experimental ones, using the Nelder Mead method[43] implemented in Scipy (version 1.10.1). The tetrahedral intermediate (Tet^-) could not be detected during the NMR experiments due to its metastable nature, confirming its very low concentration along the reaction. A similar approach was also employed for the IR-derived concentrations. However, as only the xAsp concentration could have been directly estimated from the deconvoluted spectra, we utilized the NMR quantification of the species to estimate the Asn and Suc concentrations. Specifically, at each time point, we computed the ratio between xAsp and Asn as well as the one between xAsp and Suc determined by NMR. Then, for the shared time points between IR and NMR spectroscopy, we applied the NMR-ratio to the xAsp concentration derived from IR spectroscopy, thereby obtaining an estimate of the Asn and Suc species.

To remove a possible bias due to the use of theoretical estimates as starting guesses, we test the efficiency of random combinations of the three kinetic constants associated with the formation of succinimide (k_1, k_{-1} and k_2 in Scheme 1), considering different initial pKas

(9.5, 10, 10.3 and 10.6). For each kinetic constant, 122 values within the range 10^{-5}-10^{6} were randomly assigned, obtaining approximately $1 \cdot 10^{6}$ possible combinations at each pKa. Each combination was utilized to solve the set of kinetic equations S10-S13. The combinations giving a root mean square error (RMSE) between the predicted concentrations and the NMR data without buffer at pH 8 less than 3 were further optimized, following the above-mentioned procedure. The optimized kinetic constants leading to a calculated concentration of species with an RMSE less than 0.02 and a maximum value of residuals less than $|0.05|$ for Asn and Asp and a value not higher than $|0.01|$ for Suc were regarded as potential combinations of kinetic constants. Among these, all the combinations with negative kinetic constant values were disregarded. The activation free energies, associated with the obtained kinetic constants, were estimated employing the Eyring equation (see Equation S4), using a transmission coefficient κ equal to 1 and at T=300K. A schematic illustration of the filtering process is reported in Figure 3C. The developed program (FindYourKs) might in principle be applied to any mechanism and requires only the kinetic scheme and the experimentally determined species concentrations. FindYourKs is publicly available on GitHub (https://github.com/MariaLauraDeSciscio/FindYourKs).

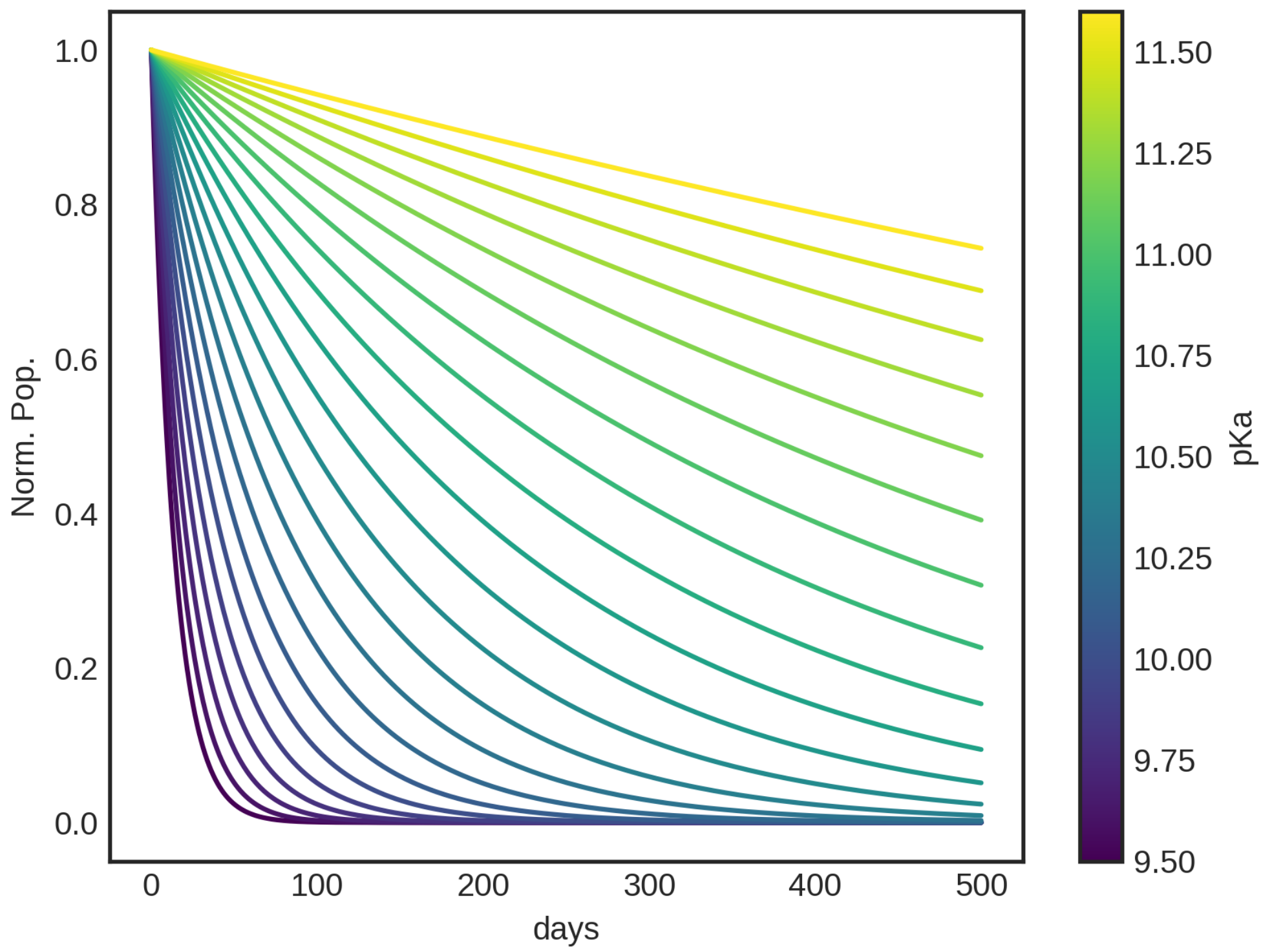

Figure S2: Theoretical estimation of the relative concentration of Asn species at pH 8, calculated by numerical integration of Equation S10, using the range of pKa comprised between the MD-PMM estimated value and the experimental error (see Methods for further details).

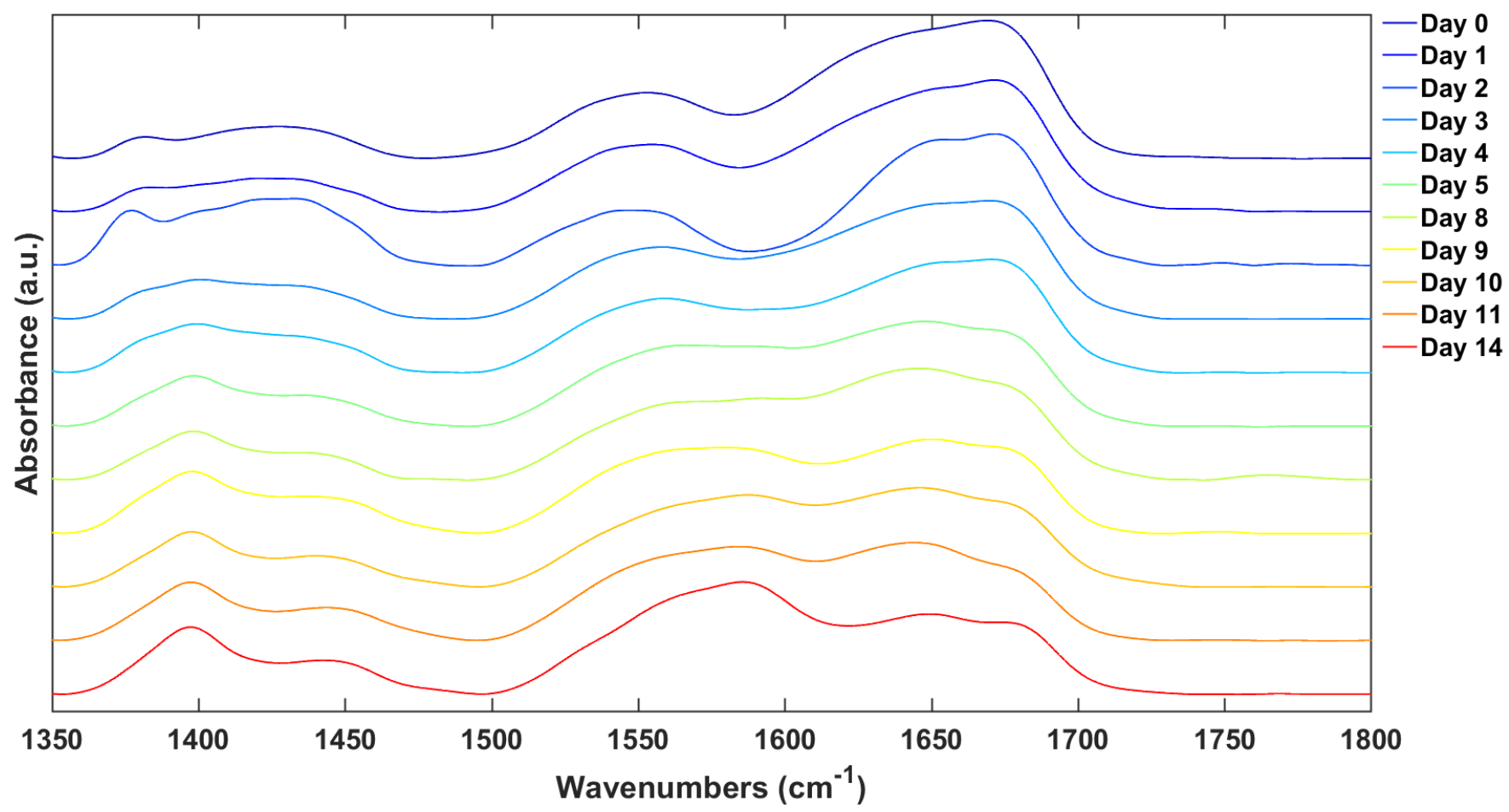

Figure S3: IR spectra of dipeptide solution over two weeks, in the spectral range between 1350-1800 cm^{-1}. IR spectra, vertically shifted to increase readability, are graphed with a scale of color from blue (Day 0) to red (Day 14). At low wavenumbers (Amide III 1350-1500 cm^{-1}), the spectrum from Day 0 (blue line in Figure S3) presents a modulated double peak with its maximum value at 1429 cm^{-1}, while the spectrum obtained on Day 14 (red line in Figure S3) shows a higher peak at 1396 cm^{-1}. At higher wavenumbers (Amide II and Amide I 1500-1800 cm^{-1}), the spectra follow a similar trend. The Day 0 spectrum shows the maximum peak at 1668 cm^{-1} in the Amide I band region, with a minimum value at 1586 cm^{-1} in the Amide II range. In contrast, the Day 14 spectrum presents its maximum value at 1586 cm^{-1} in the Amide II range; meanwhile, the spectrum intensity for the Amide I band is reduced compared to Day 0.

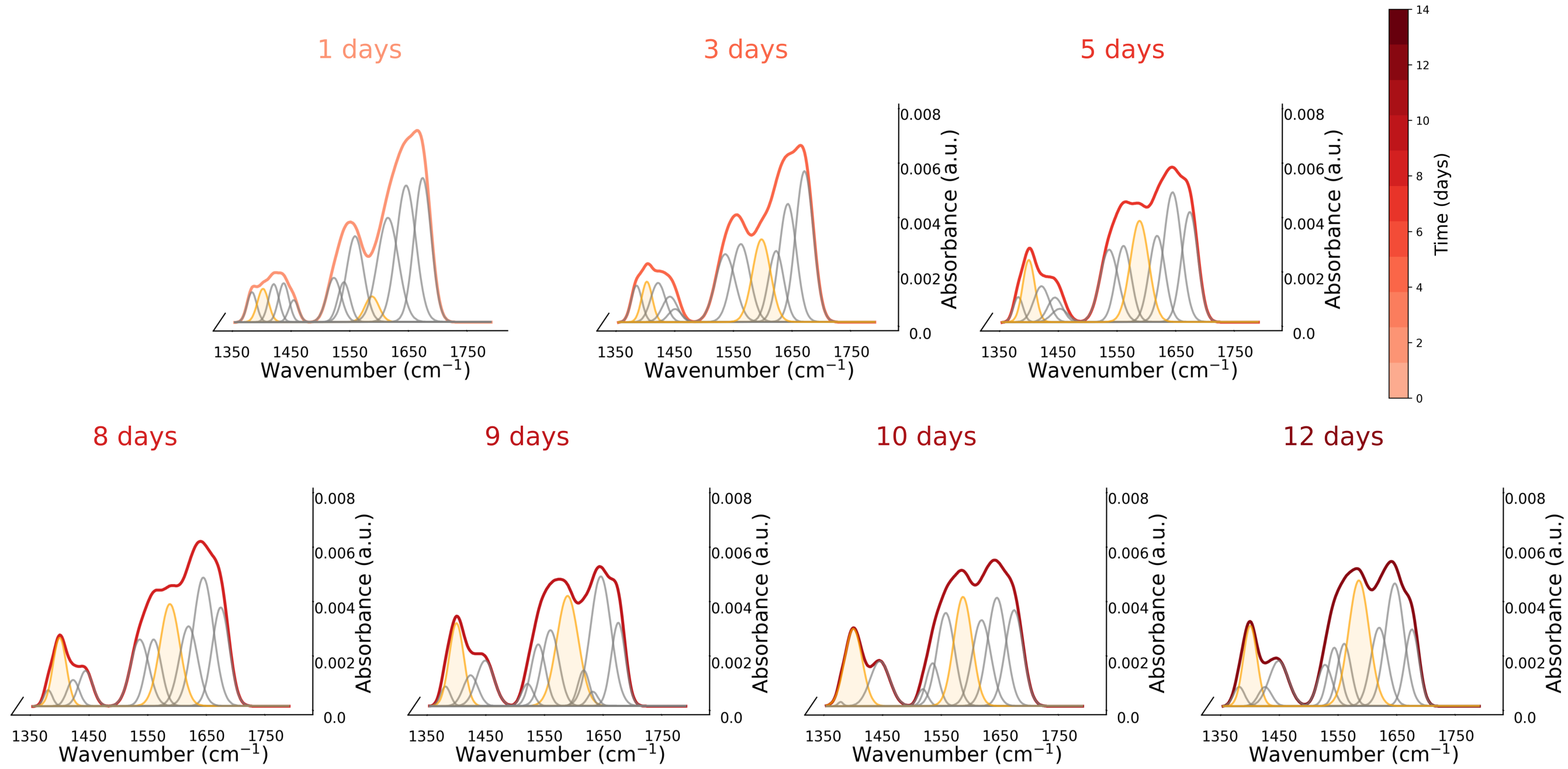

Figure S4: AsnGly Deamidation Kinetics followed by IR spectroscopy. In each panel, the experimental IR spectrum of the solution is colored according to the time-based colorbar. Below each AsnGly IR spectrum, the corresponding Gaussian deconvolution at that time point is shown as solid gray lines. The Gaussian components associated with the xAsp vibrational modes are colored and filled in pale orange.

Table S1: Area of xAsp signals (normalised by the area of xAsp at time 14 days) as a function of incubation time (pH 8; T= 298 K).

Time (days)	Area$_{xAsp}$
0	0.00
1	0.13
2	0.29
3	0.49
4	0.53
7	0.66
8	0.74
9	0.90
10	0.94
11	1.00
14	1.00

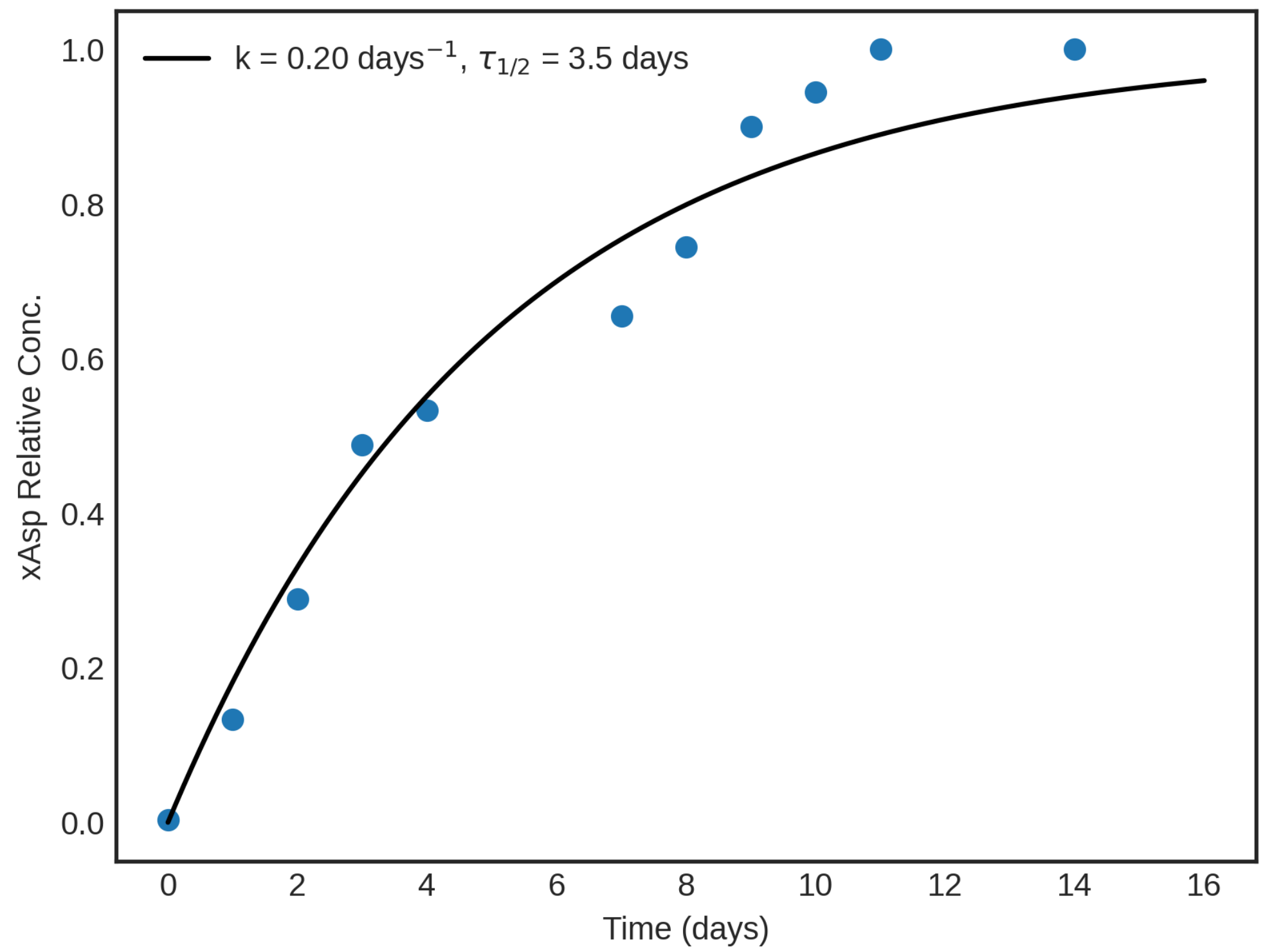

Figure S5: Relative Concentration of xAsp as a function of incubation time as estimated by the normalized areas of IR peaks (Table S1 - dots) fitted to a monoexponential equation ($y = 1 - e^{-kt}$) (continuous line).

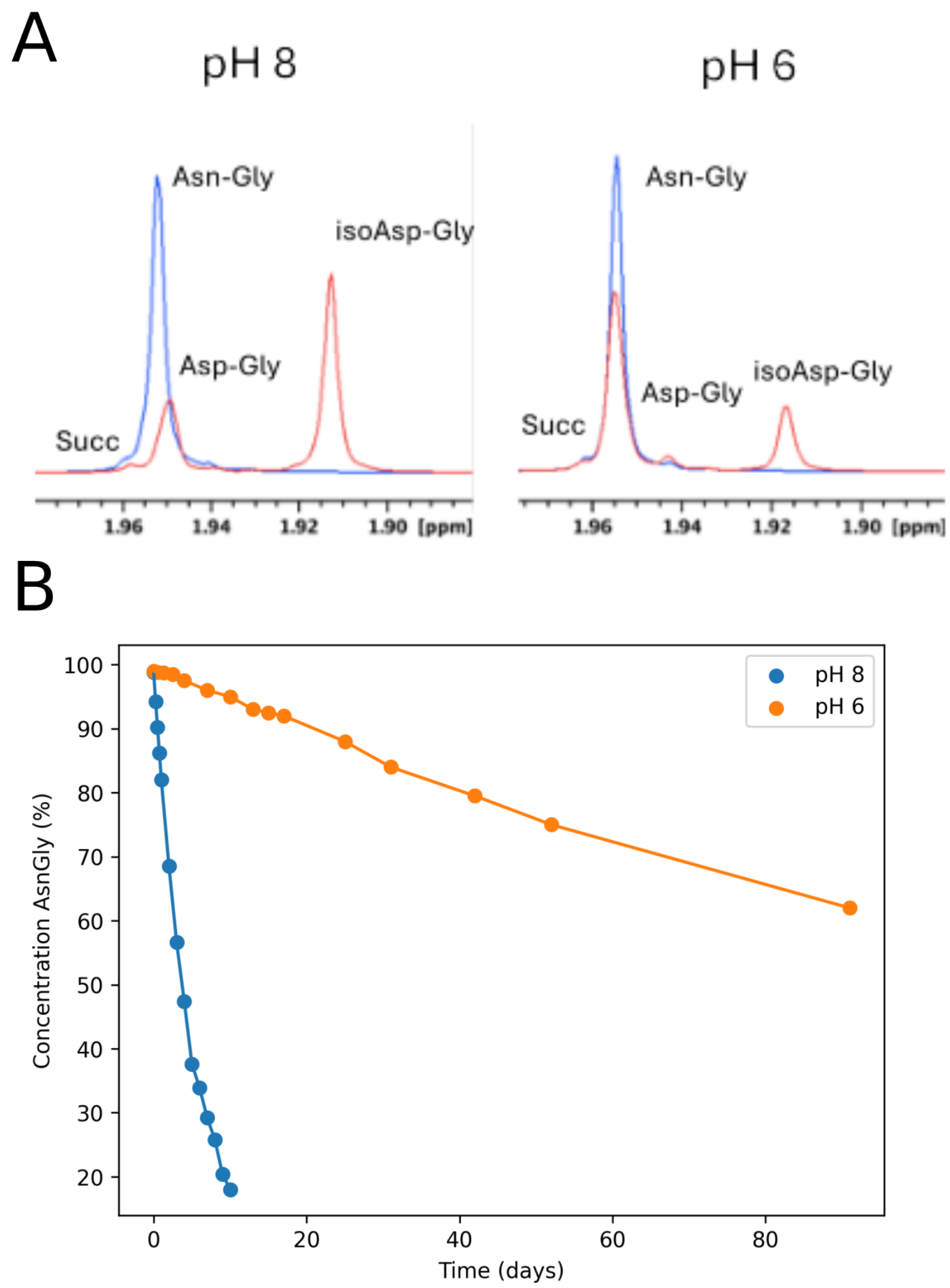

Figure S6: A) Superposition of the ^{1}H-NMR spectra showing the methyl acetate region of the AsnGly samples measured immediately after the solution preparation (blue lines) and after three months (pH6) or eight days (pH 8) (red lines) with peaks' assignment. B) AsnGly relative moles' percentage as a function of the incubation time for samples at T= 298 K and pH 6 (orange), and pH 8 (blue) in PB.

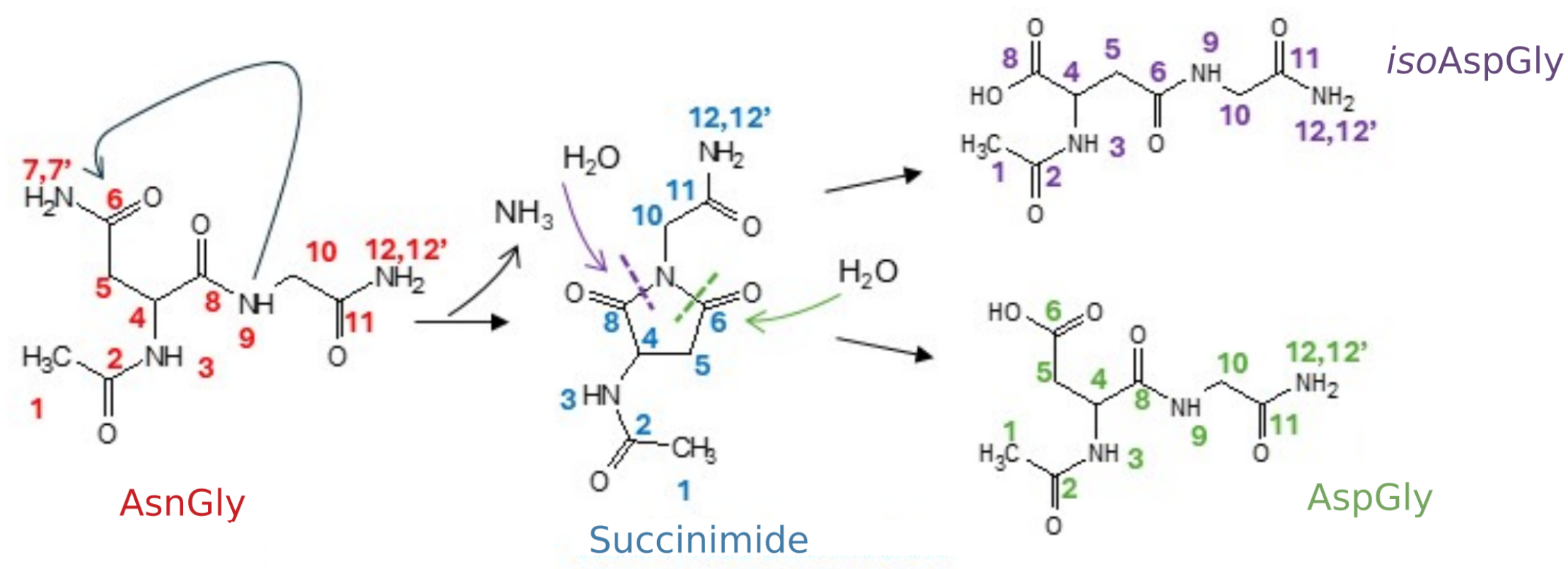

Figure S7: AsnGly deamidation reaction scheme showing the intermediate succinimide formation and its hydrolysis through which AspGly and *iso*AspGly, the final reaction products, are formed.

Table S2: ^{13}C and ^{1}H NMR chemical shifts assignments for the sample in PB at pH 6 (T= 298K); for atom numbering and color coding refer to Figure S7.

**Position**	**AsnGly**		**Succinimide**		**AspGly**		***iso*AspGly**	
	^{13}C δ	^{1}H δ	^{13}C δ	^{1}H δ	^{13}C δ	^{1}H δ	^{13}C δ	^{1}H δ
1	22.0	1.95	20.3	1.97	21.6	1.94	22.1	1.92
2	174.6	-	n.d.	-	n.d.	-	173.5	-
3	-	8.35	-	8.59	-	8.20	-	7.94
4	50.7	4.60	49.5	4.60	52.3	4.45	52.1	4.41
5	36.4	2.74/2.68	34.7	3.14/2.75	38.7	2.57/2.57	38.4	2.72/2.58
6	174.7	-	n.d.	-	177.9	-	173.8	-
7	-	7.52/6.83	-	-	-	-	-	-
8	173.6	-	n.d.	-	174.7	-	177.2	-
9	-	8.47	-	-	-	-	-	8.29
10	42.5	3.84/3.78	40.6	4.26/4.26	42.3	3.81/3.76	42.4	3.74/3.78
11	174.0	-	171.1	-	173.8	174.6	174.7	-
12	-	7.41/7.02	-	7.64/7.13	-	7.00/7.48	-	7.52/6.98

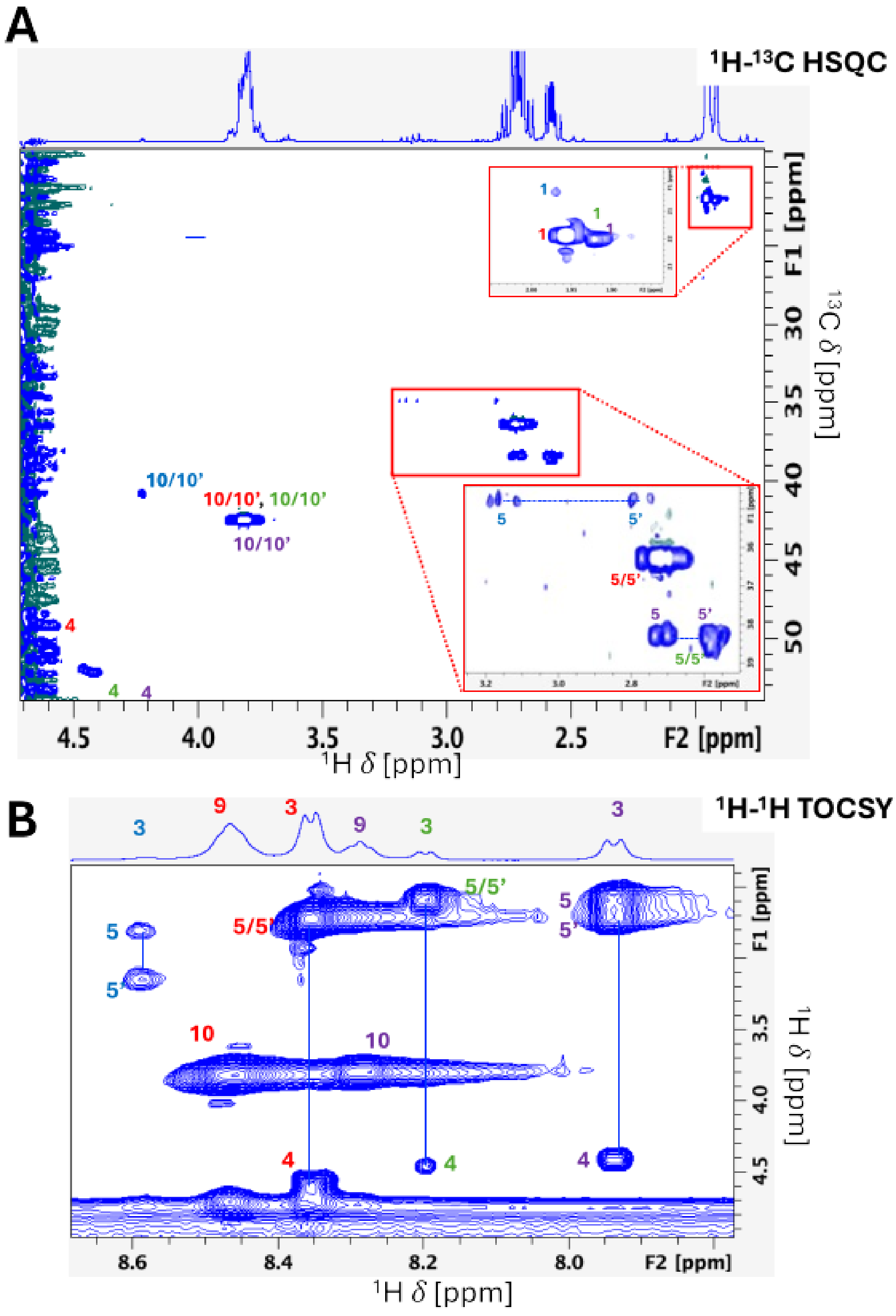

Figure S8: ^{13}C and ^{1}H resonances assignment for AsnGly, succinimide, and AspGly/*iso*AspGly deamidation products in PB (pH 6; T= 298K). ^{1}H-^{13}C HSQC (A) and amide region of the ^{1}H-^{1}H TOCSY (B) spectra recorded at the end of the reaction (52 days; 400 MHz). For the numbering and colour coding, refer to Figure S7.

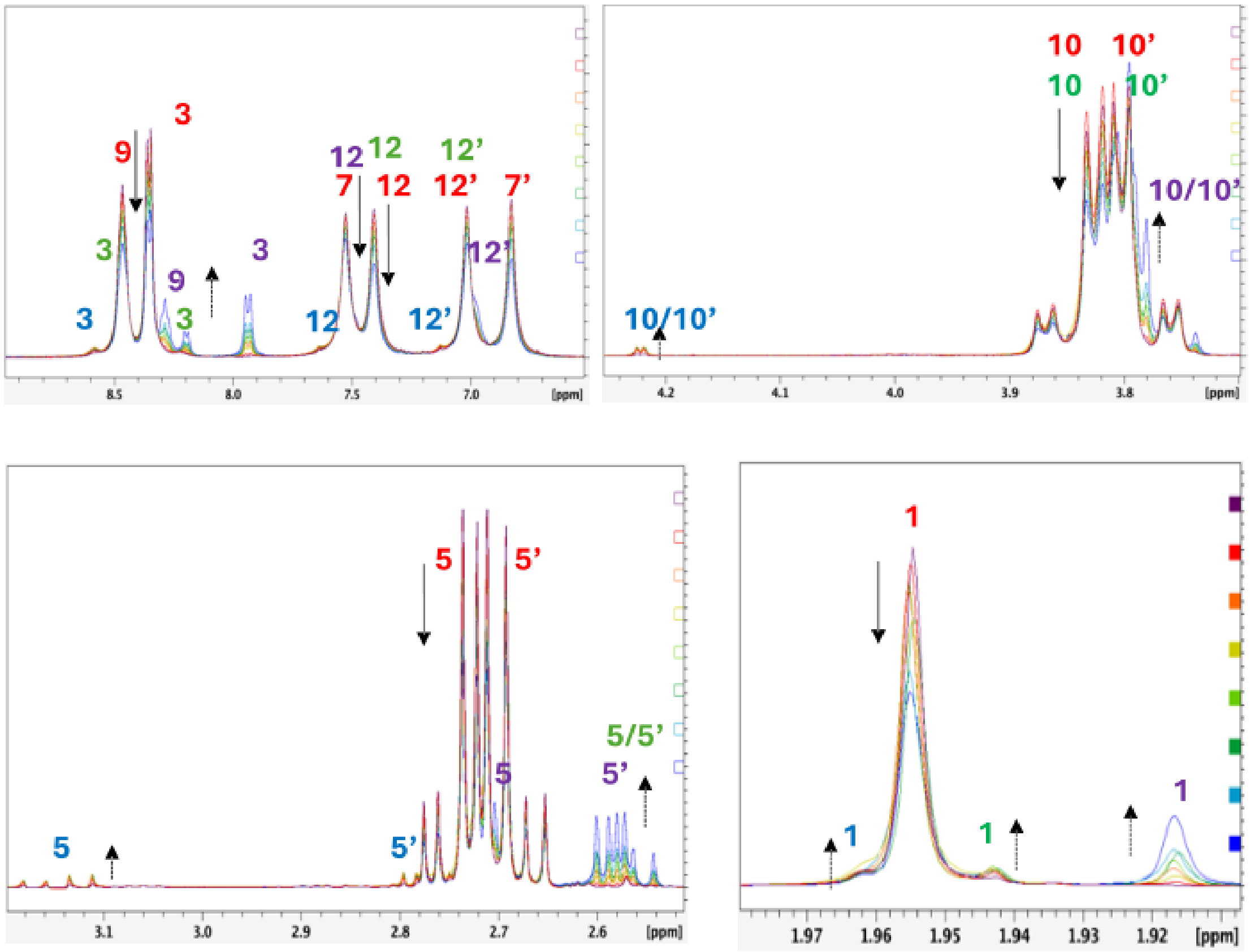

Figure S9: Diverse expansions of the superposition of the 1D NMR spectra of the AsnGly sample at pH 6 at 298 K at different reaction times (immediately and after 7, 17, 24, 31, 42, 52 and 90 days after sample preparation, from blue to violet, respectively). For the numbering and color coding, refer to Figure S7. The arrows indicate signals that increase or decrease over time.

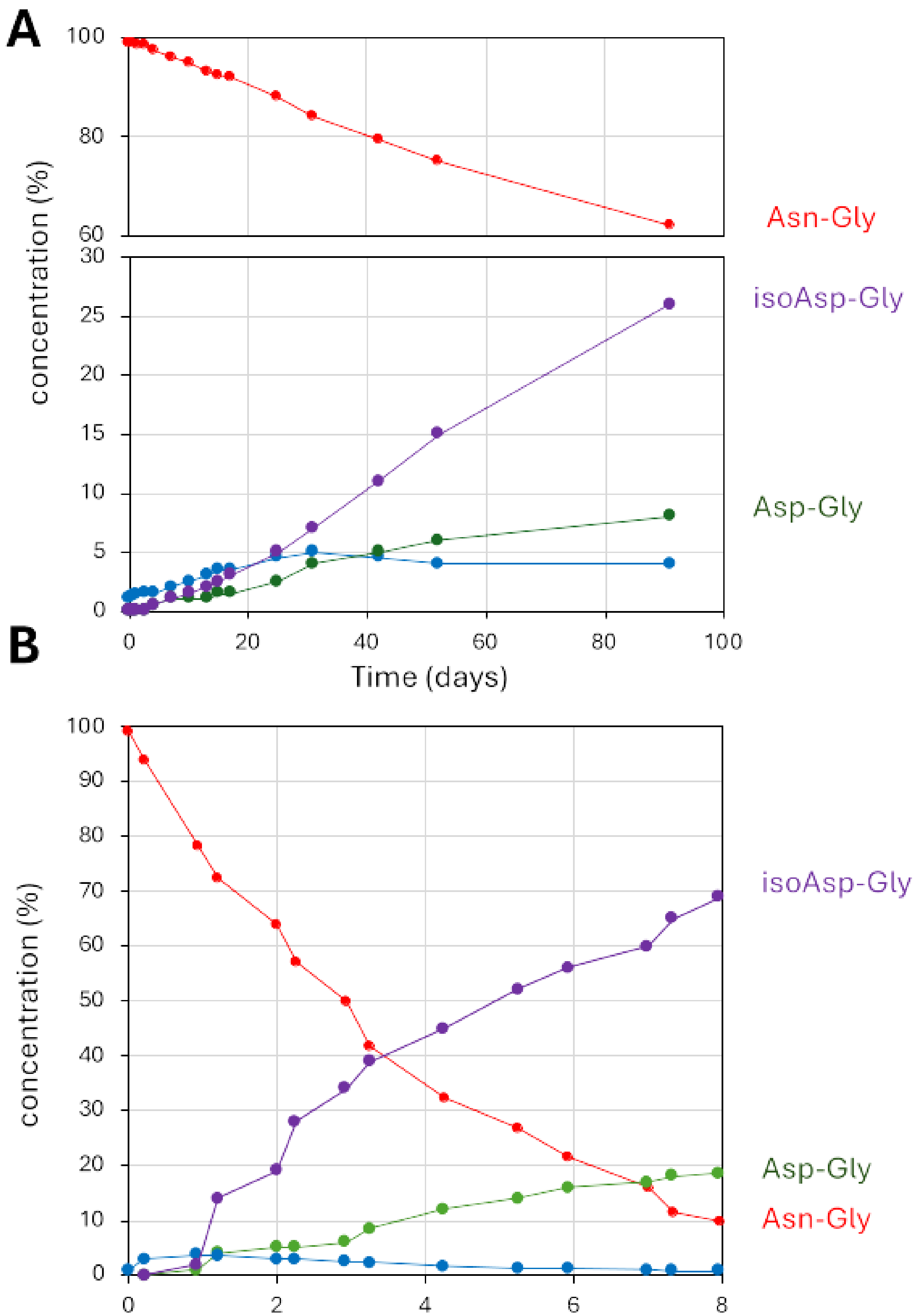

Figure S10: Relative moles percentage concentrations of AsnGly, the succinimide intermediate (blue circles), AspGly, and *iso*AspGly as a function of incubation time (T= 298 K) for samples prepared in PB at pH= 6 (panel A) and 8 (panel B).

Table S3: Relative moles % concentrations of AsnGly, the succinimide intermediate, and the AspGly and *iso*AspGly products as obtained by NMR at different time points for the deamination reaction in PB at pH 6.

**Time (days)**	**AsnGly**	**Suc**	**AspGly**	***Iso*AspGly**
0	99.0	1.0	0	0
0.50	98.8	1.2	0	0
1.25	98.7	1.3	0	0
2.50	98.5	1.5	0	0
4.00	97.5	1.5	0.5	0.5
7.00	96.0	2.0	1.0	1.0
10.00	95.0	2.5	1.0	1.5
13.00	93.0	3.0	1.0	2.0
15.00	92.5	3.5	1.5	2.5
17.00	92.0	3.5	1.5	3.0
25.00	88.0	4.5	2.5	5.0
31.00	84.0	5.0	4.0	7.0
42.00	79.5	4.5	5.0	11.0
52.00	75.0	4.0	6.0	15.0
91.00	62.0	4.0	8.0	26.0

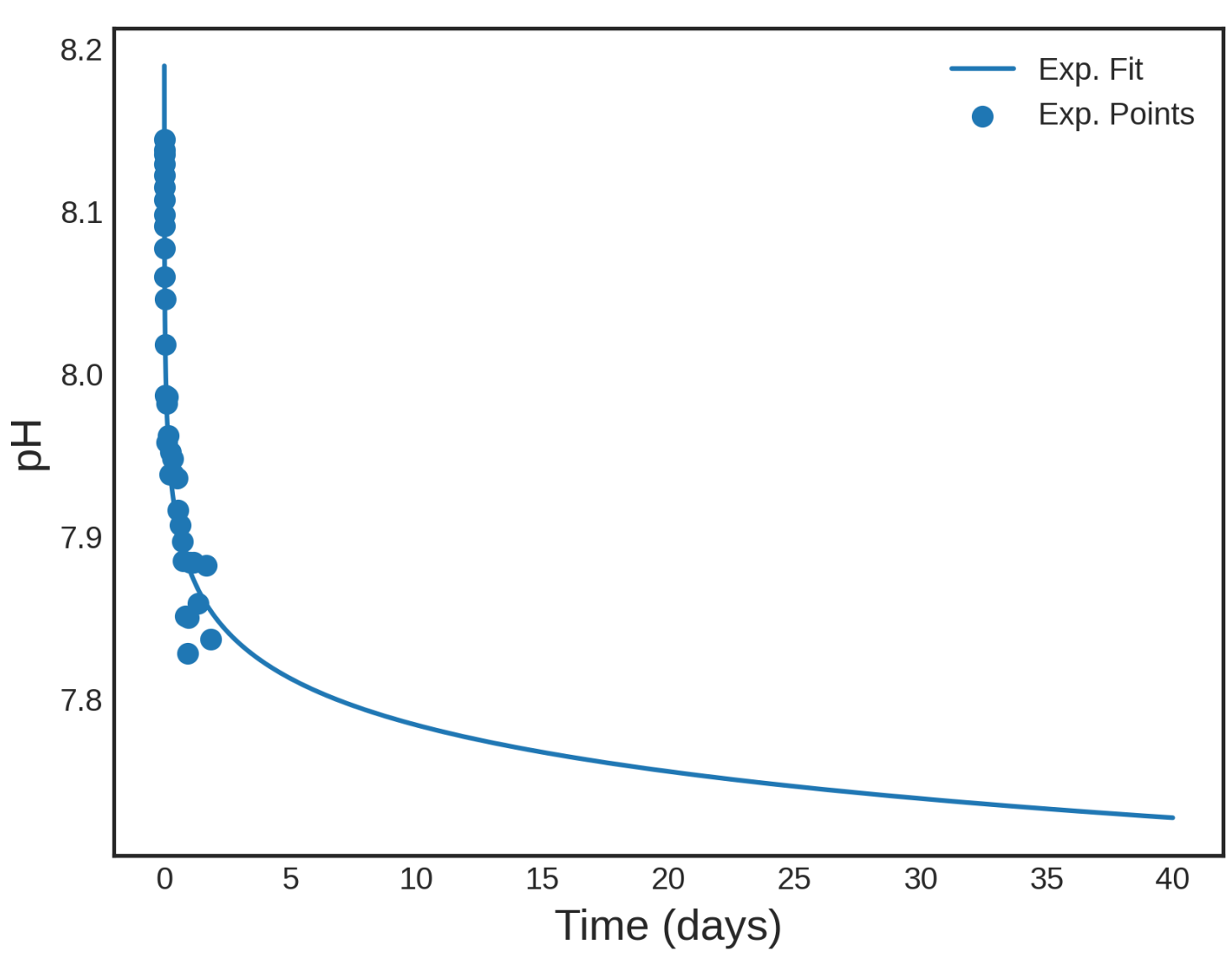

Figure S11: Temporal evolution of hydroxide ion moles, expressed as pH value, in solution along the deamidation reaction course in the absence of buffer. The solid point represents the experimental measures of pH sampled during the AsnGly deamidation reaction, while the line is the exponential decrease fit using the equation $y = a \cdot t^{-pH}$.

Table S4: Relative moles % concentrations of Asn, Suc, the AspGly and *iso*AspGly products as obtained by three independent NMR experiments (Exp1, Exp2 and Exp3) at different time points for the deamidation reaction in PB at pH 8.

**Time (days)**	**Asn**			**Suc**			**Asp**			***Iso*Asp**		
	Exp1	Exp2	Exp3	Exp1	Exp2	Exp3	Exp1	Exp2	Exp3	Exp1	Exp2	Exp3
0.00	98.80	98.80	98.80	1.20	1.20	1.20	0.00	0.00	0.00	0.00	0.00	0.00
0.25	94.20	94.30	94.40	2.50	2.60	2.60	2.40	2.40	2.30	0.90	0.70	0.60
0.50	90.20	90.30	90.40	3.00	3.00	2.90	5.10	5.10	5.20	1.70	1.60	1.60
0.75	86.20	86.40	86.40	2.90	2.90	2.90	8.20	8.20	8.40	2.70	2.50	2.50
1.00	82.00	82.50	82.40	2.90	2.80	2.80	11.40	11.30	11.80	3.70	3.40	3.50
2.00	68.50	68.50	68.50	2.20	2.50	2.40	22.50	22.40	22.70	6.80	6.60	6.70
3.00	56.60	56.70	56.60	1.80	2.10	2.10	31.80	31.70	31.80	9.80	9.50	9.60
4.00	47.40	47.20	47.00	1.60	1.60	1.70	38.90	39.40	39.40	12.10	11.80	11.90
5.00	37.60	39.30	39.30	1.40	1.40	1.40	46.70	45.70	45.50	14.30	13.60	13.60
6.00	33.90	34.10	32.90	1.10	1.20	1.40	49.90	50.30	50.10	15.10	14.40	15.40
7.00	29.20	28.90	29.70	0.90	1.00	0.90	53.60	52.70	54.20	16.30	17.40	16.70
8.00	25.80	25.30	24.50	0.90	0.80	0.90	56.20	56.70	56.90	17.10	17.20	17.90
9.00	20.40	21.00	20.60	0.60	0.70	0.60	60.50	60.20	61.60	18.50	18.10	18.60
10.00	18.00	18.50	18.00	0.60	0.50	0.60	62.30	62.30	63.10	19.10	18.70	19.10

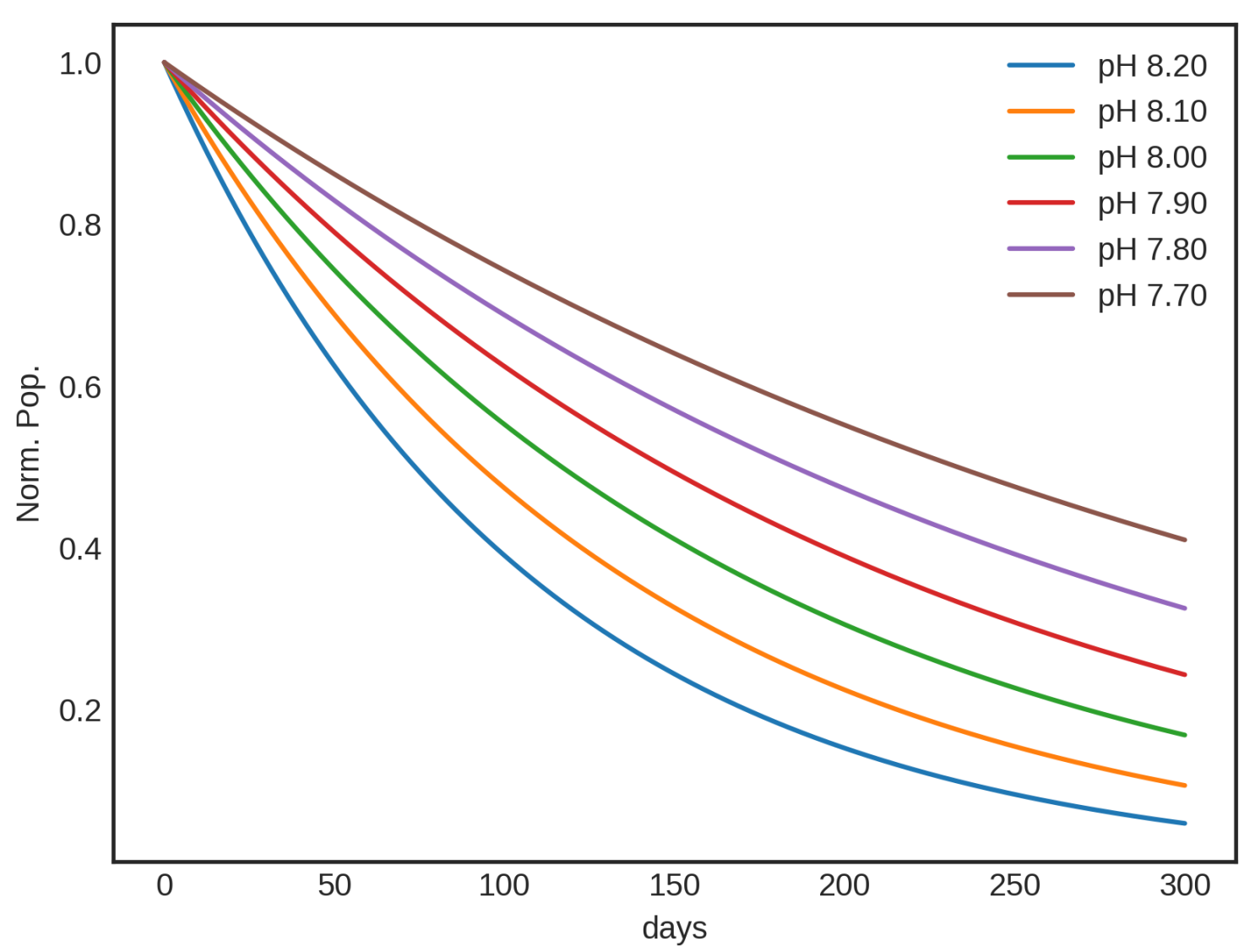

Figure S12: Simulated evolution of the relative concentration of Asn over time in the absence of buffer as a function of the measured pH variation.

Table S5: Relative moles % concentrations of Asn, Suc, the AspGly and *iso*AspGly products as obtained by three independent NMR experiments (Exp1, Exp2 and Exp3) at different time points for the deamidation reaction without buffer.

**Time (days)**	**Asn**			**Suc**			**Asp**			***Iso*Asp**		
	Exp1	Exp2	Exp3	Exp1	Exp2	Exp3	Exp1	Exp2	Exp3	Exp1	Exp2	Exp3
0.00	99.20	99.30	99.2	0.80	0.70	0.8	0.00	0.00	0.00	0.00	0.00	0.00
0.25	99.10	98.70	98.9	0.70	0.80	0.8	0.20	0.40	0.30	0.00	0.10	0.00
0.50	98.10	98.00	98.5	0.80	0.90	0.8	0.80	0.90	0.60	0.30	0.20	0.10
0.75	97.40	97.30	98	0.80	0.80	0.9	1.40	1.40	0.90	0.40	0.50	0.20
1.00	96.90	97.10	–	0.70	0.90	–	1.80	1.60	–	0.60	0.40	–
2.00	94.50	94.70	95.4	0.90	0.90	0.8	3.50	3.50	3.00	1.10	0.90	0.80
3.00	92.40	92.50	93.5	0.90	0.90	0.9	5.10	5.10	4.50	1.60	1.50	1.10
4.00	90.40	90.20	91	0.90	0.90	0.9	6.80	6.90	6.30	1.90	2.00	1.80
5.00	88.10	88.50	89	1.10	1.00	1	8.40	8.20	7.70	2.40	2.30	2.30
6.00	86.60	86.40	87.1	1.00	1.00	1	9.60	9.70	9.20	2.80	2.90	2.70
7.00	84.40	84.50	85.2	1.20	0.90	1.1	11.30	11.30	10.90	3.10	3.30	2.80
8.00	–	82.60	83.6	–	1.10	1	–	12.60	12.30	–	3.70	3.10
9.00	–	81.10	81.2	–	1.20	1.2	–	13.80	13.70	–	3.90	3.90
10.00	–	78.30	79.3	–	1.30	1.3	–	15.70	15.10	–	4.70	4.30

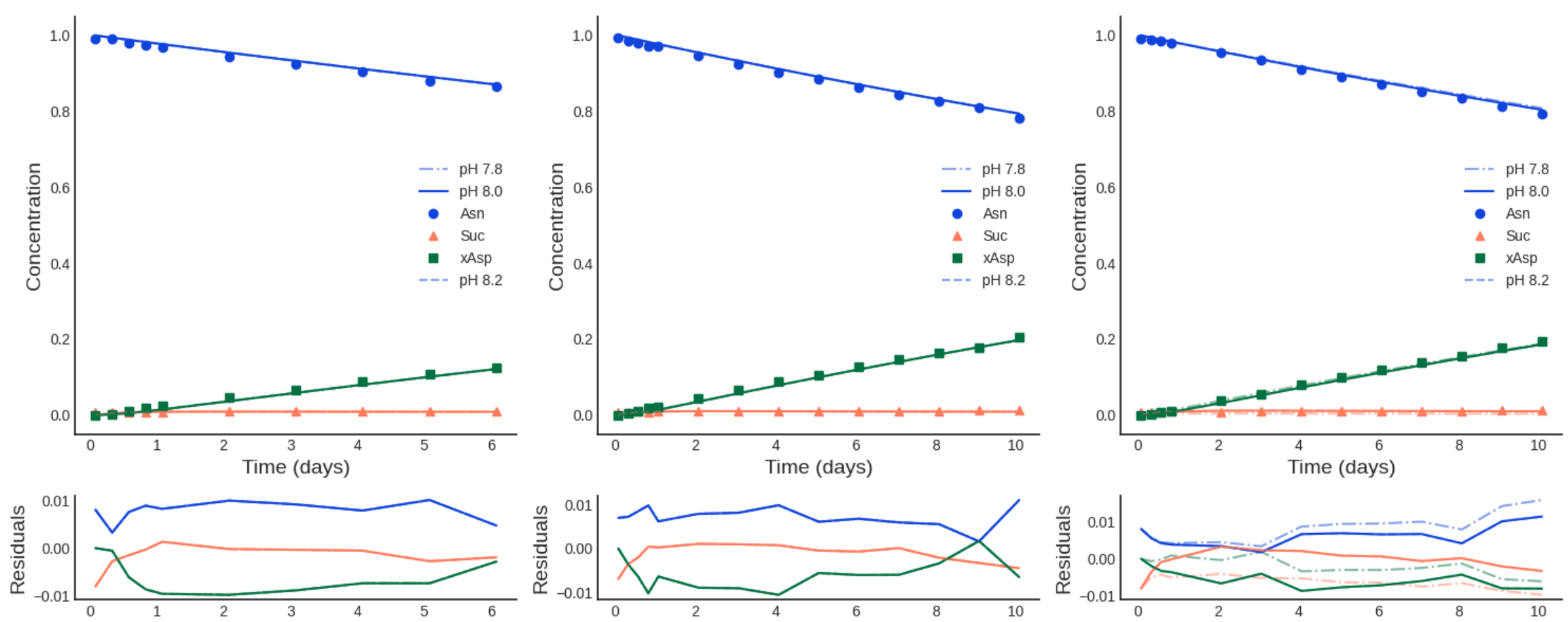

Figure S13: Evolution with time of the relative concentrations of Asn, Suc, and xAsp in NB at pH 8 as determined by NMR measurements in comparison with the normalized population variation as obtained by solving Equations S10-S13 assuming an initial pKa value of 10.6. The differences between the experimental and simulated data (residuals) are reported in the lower panel (same colors as in the main graph).

Table S6: Deamidation experimental constants estimated from NMR experiments performed in water, considering the central pH of 8.

Exp	pKa	k_1 (s^{-1})	k_1 (s^{-1})	k_2 (s^{-1})	k_3 (s^{-1})
NB 1	10.20	10.23	$3.64 \cdot 10^6$	15.43	$3.10 \cdot 10^{-5}$
NB 2	10.23	23.11	$6.30 \cdot 10^6$	12.55	$2.64 \cdot 10^{-5}$
NB 3	10.42	40.93	$5.65 \cdot 10^6$	9.31	$2.19 \cdot 10^{-5}$

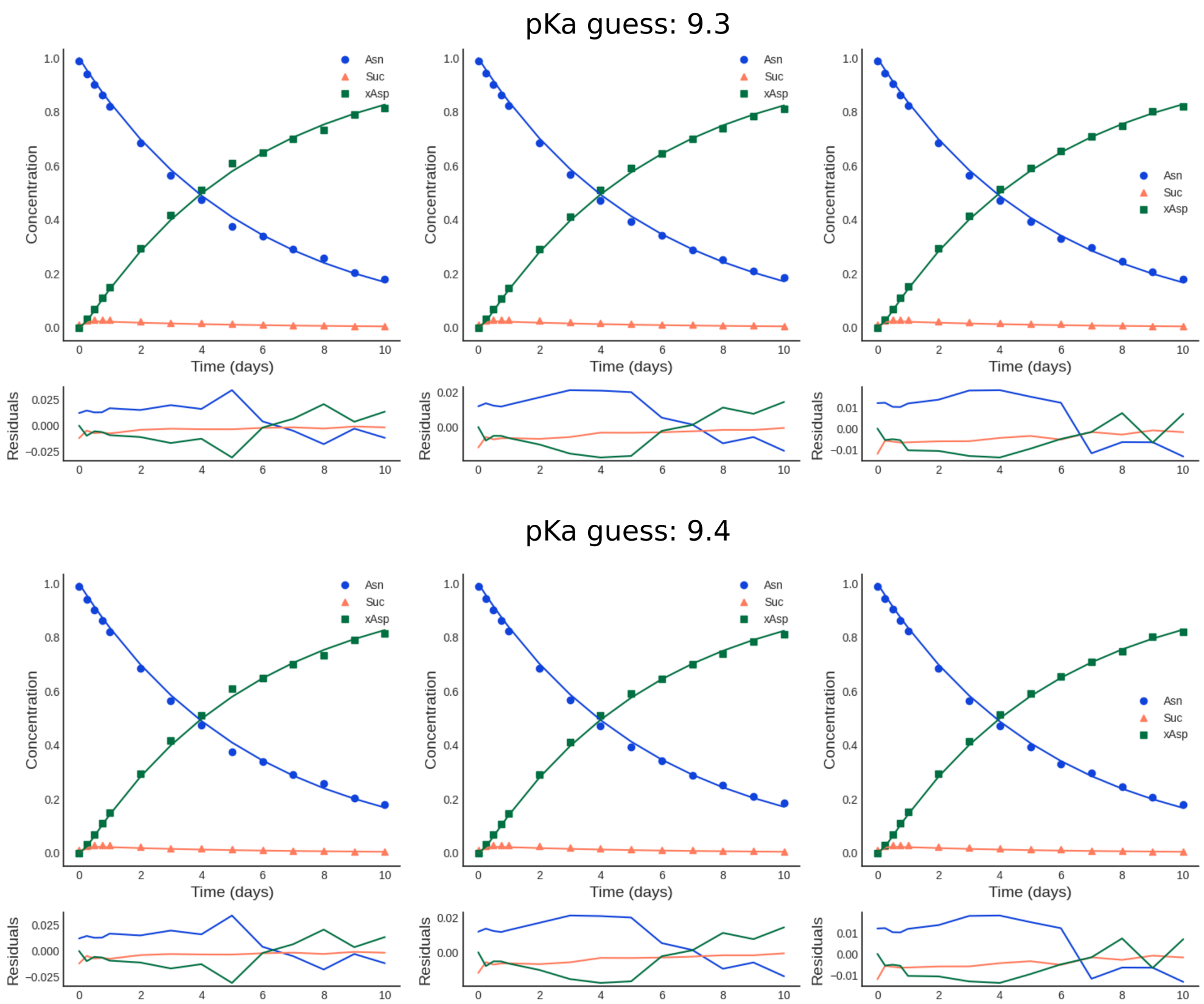

Figure S14: Evolution with time of the relative concentrations of Asn, Suc, and xAsp in PB at pH 8 as determined by NMR measurements in comparison with the normalized population variation as obtained by solving Equations S10-S13 with the kinetic constants reported in Table S7 (colored lines), assuming an initial pKa value (pKa_{in}) equal to 9.3 (top row) or 9.4 (bottom row). The differences between the experimental and simulated data (residuals) are reported in the lower panel (same colors as in the main graph). The overall RMSE is less than 0.01.

Table S7: Comparison between sets of deamidation kinetic constants, potential solutions to the set of ordinary equations describing deamidation kinetics in PB at pH 8. The same procedure for minimizing the calculated and experimental relative concentrations of species was iterated across different pKas, ranging from 9.0 to 10.6, the PMM-pKa value. The footnote *in* and *opt* indicates the initial and optimized pKa value, respectively.

Exp	pKa_{in}	pKa_{opt}	k_1 (s^{-1})	k_1 (s^{-1})	k_2 (s^{-1})	k_3 (s^{-1})	RMSE
PB 1	9.00	8.85	36.38	$1.02 \cdot 10^{7}$	6.55	$8.54 \cdot 10^{-5}$	$1.05 \cdot 10^{-2}$
PB 2	9.00	8.87	37.31	$1.03 \cdot 10^{7}$	6.57	$8.49 \cdot 10^{-5}$	$9.18 \cdot 10^{-3}$
PB 3	9.00	8.84	35.45	$1.02 \cdot 10^{7}$	6.58	$8.57 \cdot 10^{-5}$	$8.07 \cdot 10^{-3}$
PB 1	9.10	8.86	41.65	$1.11 \cdot 10^{7}$	6.33	$7.03 \cdot 10^{-5}$	$1.07 \cdot 10^{-2}$
PB 2	9.10	8.91	36.71	$9.47 \cdot 10^{6}$	6.86	$8.36 \cdot 10^{-5}$	$9.20 \cdot 10^{-3}$
PB 3	9.10	8.86	40.27	$1.08 \cdot 10^{7}$	6.41	$6.87 \cdot 10^{-5}$	$8.16 \cdot 10^{-3}$
PB 1	9.20	8.99	39.39	$8.51 \cdot 10^{6}$	6.90	$8.28 \cdot 10^{-5}$	$1.05 \cdot 10^{-2}$
PB 2	9.20	8.98	38.73	$8.65 \cdot 10^{6}$	6.96	$8.21 \cdot 10^{-5}$	$9.17 \cdot 10^{-3}$
PB 3	9.20	8.97	41.78	$9.46 \cdot 10^{6}$	7.04	$6.86 \cdot 10^{-5}$	$8.16 \cdot 10^{-3}$
PB 1	9.30	9.08	42.77	$7.40 \cdot 10^{6}$	6.76	$8.24 \cdot 10^{-5}$	$1.05 \cdot 10^{-2}$
PB 2	9.30	9.02	34.79	$7.57 \cdot 10^{6}$	7.38	$8.17 \cdot 10^{-5}$	$9.18 \cdot 10^{-3}$
PB 3	9.30	9.02	36.07	$7.50 \cdot 10^{6}$	7.14	$8.28 \cdot 10^{-5}$	$8.04 \cdot 10^{-3}$
PB 1	9.40	9.14	40.78	$6.67 \cdot 10^{6}$	7.40	$8.20 \cdot 10^{-5}$	$1.05 \cdot 10^{-2}$
PB 2	9.40	9.14	41.44	$6.76 \cdot 10^{6}$	7.25	$8.13 \cdot 10^{-5}$	$9.17 \cdot 10^{-3}$
PB 3	9.40	9.13	41.41	$6.72 \cdot 10^{6}$	7.26	$8.24 \cdot 10^{-5}$	$8.04 \cdot 10^{-3}$
PB 1	9.50	9.22	42.47	$5.42 \cdot 10^{6}$	6.87	$8.94 \cdot 10^{-5}$	$1.06 \cdot 10^{-2}$
PB 2	9.50	9.21	42.45	$5.51 \cdot 10^{6}$	6.88	$8.81 \cdot 10^{-5}$	$9.31 \cdot 10^{-3}$
PB 3	9.50	9.22	42.53	$5.42 \cdot 10^{6}$	6.87	$8.91 \cdot 10^{-5}$	$8.16 \cdot 10^{-3}$
PB 1	9.60	9.30	43.68	$4.79 \cdot 10^{6}$	7.11	$8.75 \cdot 10^{-5}$	$1.06 \cdot 10^{-2}$
PB 2	9.60	9.29	44.02	$4.85 \cdot 10^{6}$	6.96	$8.69 \cdot 10^{-5}$	$9.30 \cdot 10^{-3}$
PB 3	9.60	9.30	43.64	$4.72 \cdot 10^{6}$	7.04	$8.77 \cdot 10^{-5}$	$8.13 \cdot 10^{-3}$
PB 1	9.70	9.39	44.24	$3.86 \cdot 10^{6}$	7.02	$9.17 \cdot 10^{-5}$	$1.06 \cdot 10^{-2}$
PB 2	9.70	9.30	46.52	$4.52 \cdot 10^{6}$	6.21	$7.97 \cdot 10^{-5}$	$9.16 \cdot 10^{-3}$
PB 3	9.70	9.39	44.55	$3.86 \cdot 10^{6}$	6.93	$9.15 \cdot 10^{-5}$	$8.13 \cdot 10^{-3}$
PB 1	9.80	9.41	46.54	$3.96 \cdot 10^{6}$	7.21	$8.01 \cdot 10^{-5}$	$1.05 \cdot 10^{-2}$
PB 2	9.80	9.40	53.48	$3.66 \cdot 10^{6}$	5.55	$8.42 \cdot 10^{-5}$	$9.23 \cdot 10^{-3}$
PB 3	9.80	9.42	51.44	$3.67 \cdot 10^{6}$	6.19	$8.05 \cdot 10^{-5}$	$8.02 \cdot 10^{-3}$
PB 1	9.90	9.61	45.32	$3.11 \cdot 10^{6}$	9.11	$8.28 \cdot 10^{-5}$	$1.06 \cdot 10^{-2}$
PB 2	9.90	9.56	35.72	$3.31 \cdot 10^{6}$	10.87	$8.23 \cdot 10^{-5}$	$9.19 \cdot 10^{-3}$
PB 3	9.90	9.43	24.97	$1.75 \cdot 10^{6}$	6.17	$6.85 \cdot 10^{-5}$	$8.21 \cdot 10^{-3}$
PB 1	10.00	9.69	50.91	$2.21 \cdot 10^{6}$	7.00	$9.51 \cdot 10^{-5}$	$1.07 \cdot 10^{-2}$
PB 2	10.00	9.69	50.94	$2.24 \cdot 10^{6}$	7.03	$9.44 \cdot 10^{-5}$	$9.37 \cdot 10^{-3}$
PB 3	10.00	9.66	51.30	$2.78 \cdot 10^{6}$	8.24	$6.69 \cdot 10^{-5}$	$8.17 \cdot 10^{-3}$

Table S8: Estimated mass fraction of the Asn and Suc species from IR measurement at pH 8, as obtained by applying the NMR xAsp/Asn ratio and xAsp/Suc ratio to the xAsp IR estimation (see Methods for further details).

Time (days)	Asn	Suc	xAsp
0.0	0.99	0.01	0.00
1.0	0.80	0.04	0.16
2.0	0.62	0.03	0.35
3.0	0.39	0.02	0.59
4.0	0.34	0.02	0.64
7.0	0.20	0.01	0.79
8.0	0.10	0.01	0.89

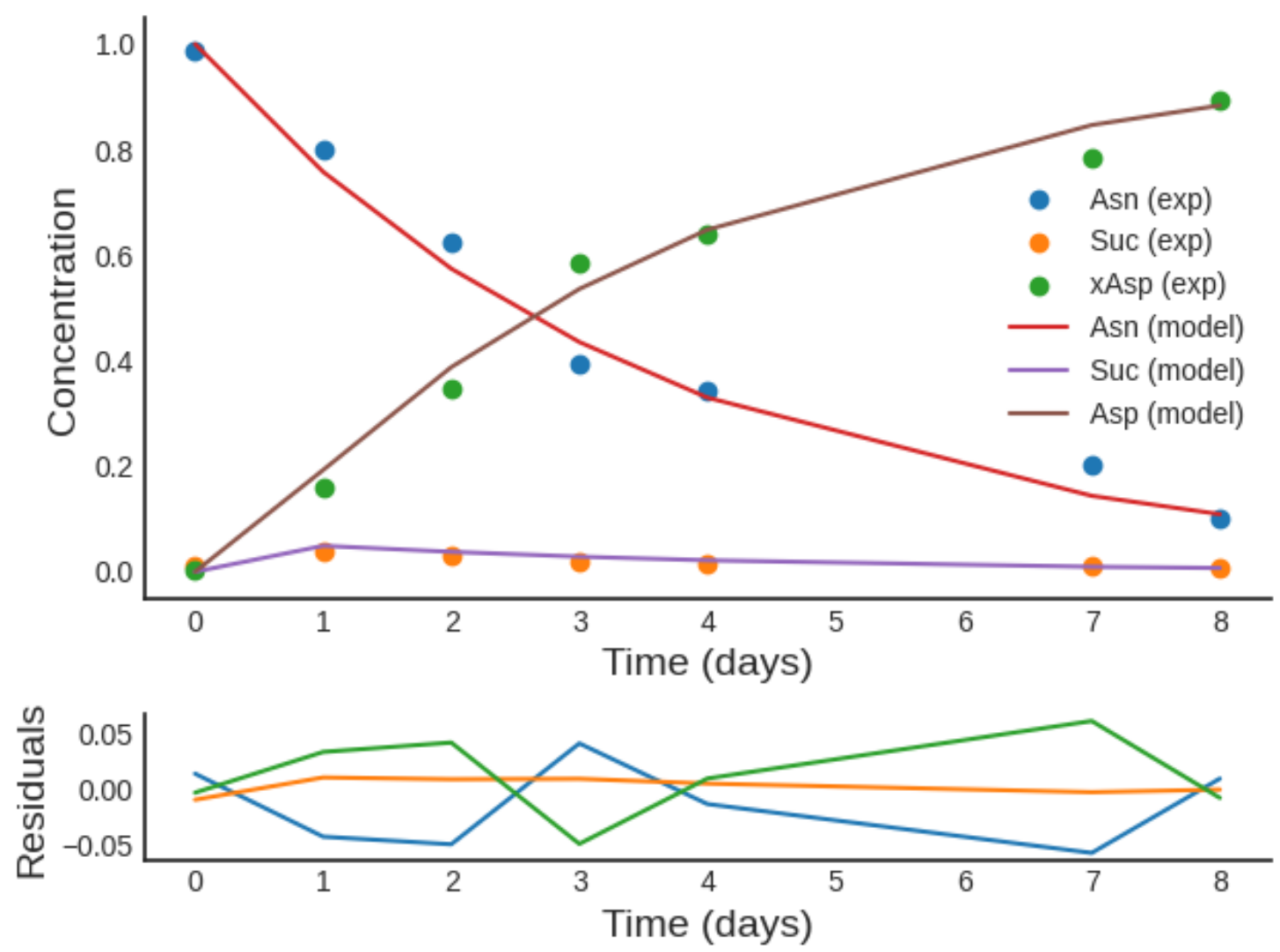

Figure S15: Single-step kinetic constants estimation (upper panel; filled circles) obtained by the fitting of IR relative concentrations of species (Table S1) in PB at pH 8 and T= 298 K. Theoretical kinetic constants utilized as the initial guesses (see Methods) resulted in a satisfactory representation of the IR concentration trend, with an RMSE lower than 0.025. The simulated variation of relative populations is shown as solid lines. The differences between the experimental data and the mathematical simulation of the concentration changes (residuals) are reported in the lower panel (same colors as in the upper panel).

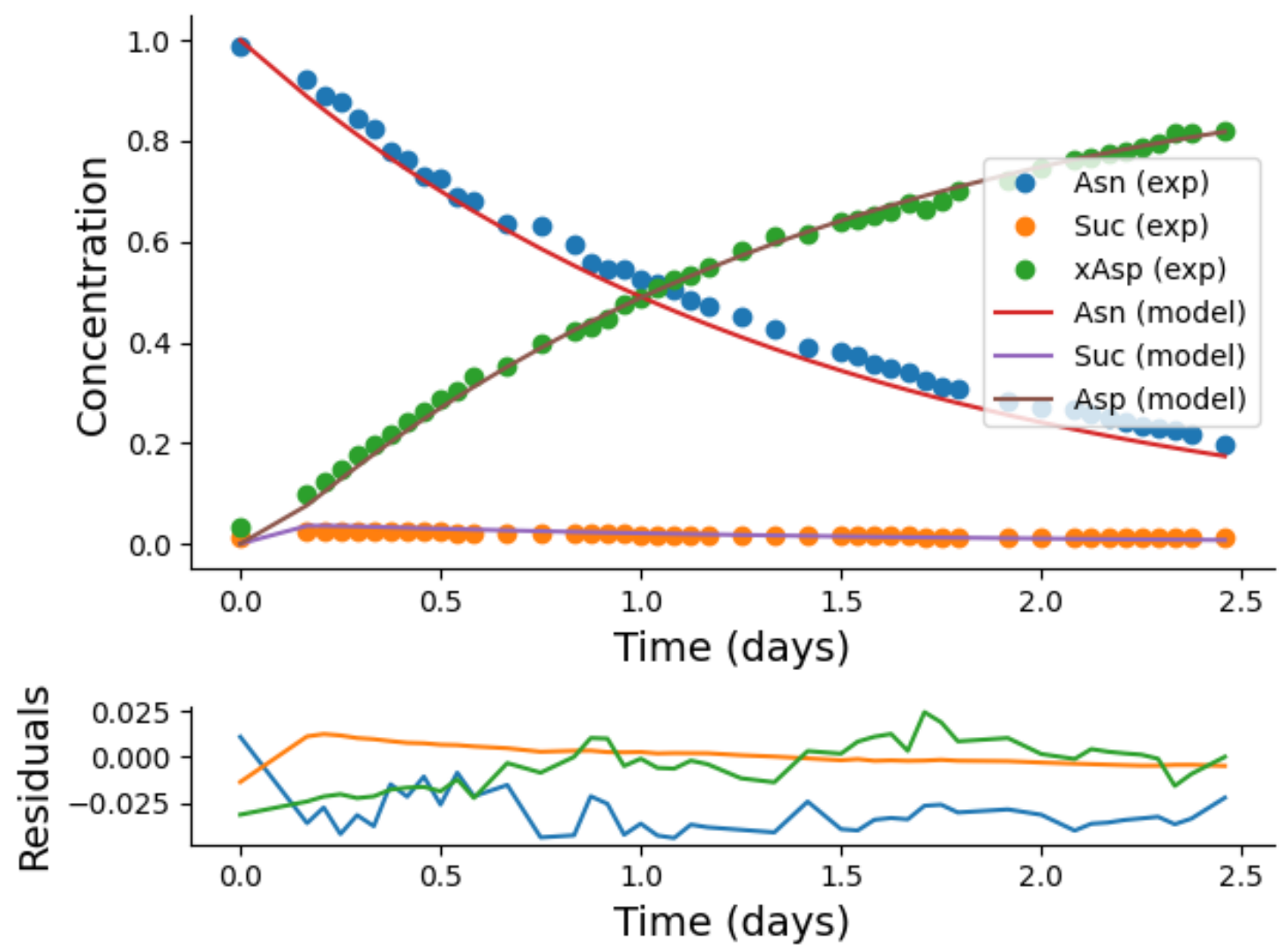

Figure S16: Ac-NGAA-NH_2 deamidation in PB at pH 7.4 and T=310K, as monitored through NMR spectroscopy by Pilhal and coworkers.[44] Experimental data (solid points) were automatically digitized using WebPlotDigitizer; data were then prepared using the same procedure adopted for the AsnGly NMR data described in the Method section. The kinetic constants utilized as an initial guess were estimated through Eyring's equation using T=310K. The optimized kinetic constants extracted, resulting in the variation of species concentration shown as solid lines and providing an RMSE lower than 0.018, are: K $= 10^{-2.25}$ (pKa = 9.65 at T=310K), $k_1 = 17.19$, $k_{-1} = 7.1 \cdot 10^4$, $k_2 = 5.34$ and $k_3 = 2.0 \cdot 10^{-4}$.

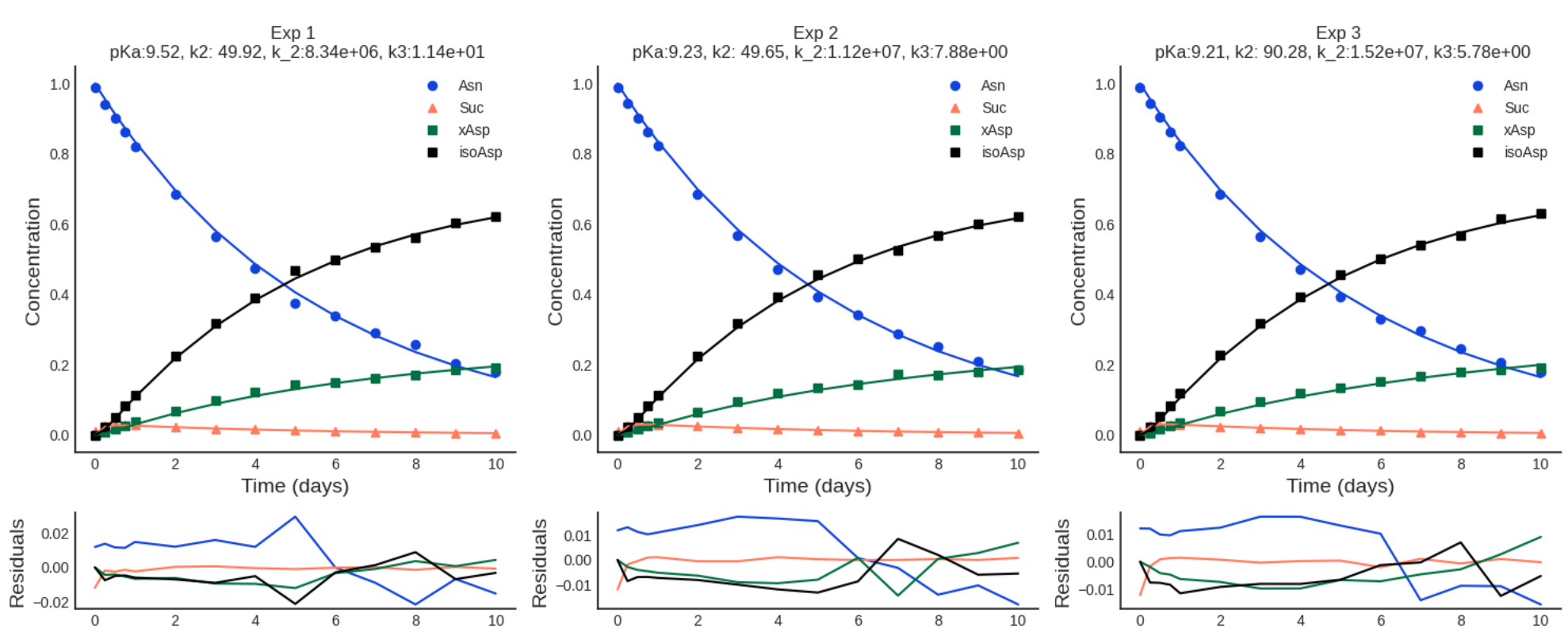

Figure S17: Comprehensive AsnGly deamidation to Asp and *iso*Asp, considering a complete reversible mechanism for succinimide hydrolysis, using eqs. S10-S11 and S14-S16. The kinetic constants associated with the forward and backward reactions of succinimide hydrolysis into Asp and *iso*Asp were retrieved from the work of Geiger and Clarke,[40] in which these constants were estimated using NMR spectroscopy in PB at pH 7.4 ($k_4 = 1.79 \cdot 10^{-5}$, $k_{-4} = 1.65 \cdot 10^{-7}$, $k_5 = 6.56 \cdot 10^{-5}$, $k_{-5} = 1.65 \cdot 10^{-7}$, all in s^{-1}). The optimized kinetic constants providing an average RMSE minor than 0.15 (calculated on all the species) are: K $= (5.03 \pm 1.72) \cdot 10^{-2}$, $k_1 = (6.33 \pm 2.34) \cdot 10$, $k_{-1} = (1.16 \pm 0.35) \cdot 10^7$, $k_2 = (8.37 \pm 2.87) \cdot 10$ s^{-1}.

Table S9: Average free energies (kcal/mol) barriers for the two-step succinimide formation associated with the kinetic constants obtained at the end of the filtering process imposing K=0.1 (pKa= 9). The corresponding MD-PMM free energy barriers are reported for comparison. The error on these values is less than 0.1 percent.

	ΔA_1	ΔA_{-1}	ΔA_2	Nr. of solutions
Model pKa 9.5	17.76± 0.05	11.06 ± 0.24	17.66 ± 0.22	33
Model pKa 10	20.17 ± 2.33	15.27 ± 3.32	18.74 ± 3.24	9093
Model pKa 10.3	17.74 ± 0.17	12.15 ± 0.18	17.67 ± 0.16	320
Model pKa 10.6	17.62 ± 0.51	12.37 ± 0.53	17.63 ± 0.36	613
MD-PMM	16.06	9.90	17.36	-

References

(1) Nardi, A. N.; Olivieri, A.; Amadei, A.; Salvio, R.; D'Abramo, M. Modelling Complex Bimolecular Reactions in a Condensed Phase: The Case of Phosphodiester Hydrolysis. *Molecules* **2023**, *28*, 2152.

(2) Nardi, A. N.; Olivieri, A.; D'Abramo, M.; Salvio, R. Unveiling the Cleavage Mechanism of an RNA Model Compound on the whole pH Scale: Computations Meet Experiments in the Determination of Reaction Rates. *ChemPhysChem* **2024**, e202300873.

(3) Capone, M.; Sirohiwal, A.; Aschi, M.; Pantazis, D. A.; Daidone, I. Alternative Fast and Slow Primary Charge-Separation Pathways in Photosystem II. *Angew Chem. Int. Ed. Engl.* **2023**, *62*, e202216276.

(4) De Sciscio, M. L.; Nardi, A. N.; Centola, F.; Rossi, M.; Guarnera, E.; D'Abramo, M. Molecular Modeling of the Deamidation Reaction in Solution: A Theoretical–Computational Study. *J. Phys. Chem. B* **2023**, *127*, 9550–9559.

(5) De Sciscio, M. L.; D'Annibale, V.; D'Abramo, M. Theoretical Evaluation of Sulfur-Based Reactions as a Model for Biological Antioxidant Defense. *Int. J. Mol. Sci.* **2022**, *23*, 14515.

(6) De Sciscio, M. L.; Centola, F.; Saporiti, S.; D'Abramo, M. Dissecting Methionine Oxidation by Hydrogen Peroxide in Proteins: Thermodynamics, Kinetics, and Susceptibility Descriptors. *J. Chem. Inf. Model* **2025**, *65*, 749–761.

(7) Zanetti-Polzi, L.; Del Galdo, S.; Daidone, I.; D'Abramo, M.; Barone, V.; Aschi, M.; Amadei, A. Extending the perturbed matrix method beyond the dipolar approximation: comparison of different levels of theory. *Phys. Chem. Chem. Phys* **2018**, *20*, 24369–24378.

(8) Chen, C. G.; Nardi, A. N.; Amadei, A.; D'Abramo, M. PyMM: An Open-Source Python Program for QM/MM Simulations Based on the Perturbed Matrix Method. *J. Chem. Theory Comput.* **2023**, *19*, 33–41.

(9) Amadei, A.; D'Alessandro, M.; Aschi, M. Statistical Mechanical Modeling of Chemical Reactions in Complex Systems: The Reaction Free Energy Surface. *J. Phys. Chem. B* **2004**, *108*, 16250–16254.

(10) Amadei, A.; D'Abramo, M.; Daidone, I.; D'Alessandro, M.; Nola, A. D.; Aschi, M. Statistical mechanical modelling of chemical reactions in complex systems: the kinetics of the Haem carbon monoxide binding–unbinding reaction in Myoglobin. *Theor. Chem. Acc.* **2007**, *117*, 637–647.

(11) Peters, B. In *Reaction Rate Theory and Rare Events Simulations*; Peters, B., Ed.; Elsevier: Amsterdam, 2017; pp 227–271.

(12) Zanetti-Polzi, L.; Daidone, I.; Amadei, A. Fully Atomistic Multiscale Approach for pKa Prediction. *J. Phys. Chem. B* **2020**, *124*, 4712–4722.

(13) D'Annibale, V.; Fracassi, D.; Marracino, P.; D'Inzeo, G.; D'Abramo, M. Effects of Environmental and Electric Perturbations on the pKa of Thioredoxin Cysteine 35: A Computational Study. *Molecules* **2022**, *27*, 6454.

(14) Albrecht, M.; Rice, C. A.; Suhm, M. A. Elementary Peptide Motifs in the Gas Phase: FTIR Aggregation Study of Formamide, Acetamide, N-Methylformamide, and N-Methylacetamide. *J. Phys. Chem. A* **2008**, *112*, 7530–7542.

(15) Muckerman, J. T.; Skone, J. H.; Ning, M.; Wasada-Tsutsui, Y. Toward the accurate calculation of pKa values in water and acetonitrile. *Biochim. Biophys. Acta (BBA) - Bioenergetics* **2013**, *1827*, 882–891.

(16) Frisch, M. J. et al. Gaussian˜16 Revision C.01. 2016.

(17) Kelemen, A. A.; Perczel, A.; Horvath, D.; Jakli, I. Amide isomerization pathways: Electronic and structural background of protonation- and deprotonation-mediated cis-trans interconversions. *J. Chem. Phys.* **2023**, *159*, 154301.

(18) Hanwell, M. D.; Curtis, D. E.; Lonie, D. C.; Vandermeersch, T.; Zurek, E.; Hutchison, G. R. Avogadro: an advanced semantic chemical editor, visualization, and analysis platform. *Journal of Cheminformatics* **2012**, *4*, 17.

(19) Sousa da Silva, A. W.; Vranken, W. F. ACPYPE - AnteChamber PYthon Parser interfacE. *BMC Research Notes* **2012**, *5*, 367.

(20) Abraham, M. J.; Murtola, T.; Schulz, R.; Pall, S.; Smith, B., J.C. amd Hess; Lindahl, E. GROMACS: High performance molecular simulations through multi-level parallelism from laptops to supercomputers. *SoftwareX* **2015**, *1-2*, 19–25.

(21) Del Galdo, S.; Marracino, P.; D'Abramo, M.; Amadei, A. In silico characterization of protein partial molecular volumes and hydration shells. *Phys. Chem. Chem. Phys.* **2015**, *17*, 31270–31277.

(22) De Sciscio, M. L.; Nardi, A. N.; Parisi, G.; Bulfaro, G.; Costanzo, A.; Gugole, E.; Exertier, C.; Freda, I.; Savino, C.; Vallone, B.; Montemiglio, L. C.; D'Abramo, M. Effect

of Salts on the Conformational Dynamics of the Cytochrome P450 OleP. *Molecules* **2023**, *28*, 832.

(23) Bussi, G.; Donadio, D.; Parrinello, M. Canonical sampling through velocity rescaling. *J. Chem. Phys.* **2007**, *126*, 014101.

(24) Darden, T.; York, D.; Pedersen, L. G. Particle mesh Ewald: An N-log(N) method for Ewald sums in large systems. *J. Chem. Phys.* **1993**, *98*, 10089–10092.

(25) Essmann, U.; Perera, L.; Berkowitz, M. L.; Darden, T.; Lee, H.; Pedersen, L. G. A smooth particle mesh Ewald method. *J. Chem. Phys.* **1995**, *103*, 8577–8593.

(26) Páll, S.; Hess, B. A flexible algorithm for calculating pair interactions on SIMD architectures. *Computer Physics Communications* **2013**, *184*, 2641–2650.

(27) Decouzon, M.; Exner, O.; Gal, J. F.; Maria, P. C. The gas-phase acidity and the acidic site of acetohydroxamic acid: a FT-ICR study. *J. Org. Chem.* **1990**, *55*, 3980–3981.

(28) Kelly, C. P.; Cramer, C. J.; Truhlar, D. G. Aqueous Solvation Free Energies of Ions and IonWater Clusters Based on an Accurate Value for the Absolute Aqueous Solvation Free Energy of the Proton. *J. Phys. Chem. B* **2006**, *110*, 16066–16081.

(29) Piccirilli, F.; Tardani, F.; D'Arco, A.; Birarda, G.; Vaccari, L.; Sennato, S.; Casciardi, S.; Lupi, S. Infrared Nanospectroscopy Reveals DNA Structural Modifications upon Immobilization onto Clay Nanotubes. *Nanomaterials* **2021**, *11*, 1103.

(30) D'Arco, A.; Di Fabrizio, M.; Mancini, T.; Mosetti, R.; Macis, S.; Tranfo, G.; Della Ventura, G.; Marcelli, A.; Petrarca, M.; Lupi, S. Secondary Structures of MERS-CoV, SARS-CoV, and SARS-CoV-2 Spike Proteins Revealed by Infrared Vibrational Spectroscopy. *Int. J. Mol. Sci.* **2023**, *24*, 9550.

(31) Mancini, T.; Macis, S.; Mosetti, R.; Luchetti, N.; Minicozzi, V.; Notargiacomo, A.; Pea, M.; Marcelli, A.; Ventura, G. D.; Lupi, S.; D'Arco, A. Infrared Spectroscopy of

SARS-CoV-2 Viral Protein: from Receptor Binding Domain to Spike Protein. *Adv. Sci.* **2024**, *11*, 2400823.

(32) Fardelli, E.; D'Arco, A.; Lupi, S.; Billi, D.; Moeller, R.; Guidi, M. C. Spectroscopic evidence of the radioresistance of *Chroococcidiopsis* biosignatures: A combined Raman, FT-IR and THz-TDs spectroscopy study. *Spectrochimica Acta Part A: Molecular and Biomolecular Spectroscopy* **2023**, *288*, 122148.

(33) Barth, A. Infrared spectroscopy of proteins. *Biochim. Biophys. Acta (BBA) - Bioenergetics* **2007**, *1767*, 1073–1101.

(34) Yang, H.; Yang, S.; Kong, J.; Dong, A.; Yu, S. Obtaining information about protein secondary structures in aqueous solution using Fourier transform IR spectroscopy. *Nat Protoc* **2015**, *10*, 382–396.

(35) Barth, A.; Zscherp, C. What vibrations tell about proteins. *Q. Rev. Biophys.* **2002**, *35*, 369–430.

(36) Rau, J. V. et al. Strontium Substituted Tricalcium Phosphate Bone Cement: Short and Long-Term Time-Resolved Studies and In Vitro Properties. *Advanced Materials Interfaces* **2022**, *9*, 2200803.

(37) Rieppo, L.; Saarakkala, S.; Närhi, T.; Helminen, H. J.; Jurvelin, J. S.; Rieppo, J. Application of second derivative spectroscopy for increasing molecular specificity of fourier transform infrared spectroscopic imaging of articular cartilage. *Osteoarthritis and Cartilage* **2012**, *20*, 451–459.

(38) Capasso, S. Kinetics and Mechanism of the Reversible lsomerization of Aspartic Acid Residues in Tetrapeptides. *J. Chem. Soc., Perkin Trans. 2* **1995**, *2*, 437–442.

(39) Patel, K.; Borchardt, R. T. Chemical pathways of peptide degradation. III. Effect of pri-

mary sequence on the pathways of deamidation of asparaginyl residues in hexapeptides. *Pharm. Res.* **1990**, *7*, 787–793.

(40) Geiger, T.; Clarke, S. Deamidation, isomerization, and racemization at asparaginyl and aspartyl residues in peptides. Succinimide-linked reactions that contribute to protein degradation. *J. Biol. Chem.* **1987**, *262*, 785–794.

(41) Kaliman, I.; Nemukhin, A.; Varfolomeev, S. Free Energy Barriers for the N-Terminal Asparagine to Succinimide Conversion: Quantum Molecular Dynamics Simulations for the Fully Solvated Model. *J. Chem. Theory Comput.* **2010**, *6*, 184–189.

(42) Xie, M.; Vander Velde, D.; Morton, M.; Borchardt, R. T.; Schowen, R. L. pH-Induced Change in the Rate-Determining Step for the Hydrolysis of the Asp/Asn-Derived Cyclic-Imide Intermediate in Protein Degradation. *J. Am. Chem. Soc.* **1996**, *118*, 8955–8956.

(43) Gao, F.; Han, L. Implementing the Nelder-Mead simplex algorithm with adaptive parameters. *Comput Optim Appl* **2012**, *51*, 259–277.

(44) Pilhál, F.; Jákli, I.; Keszei, E.; Láng, A.; Perczel, A. Kinetic, thermodynamic, and ab initio insights of AsnGly isomerisation as a ticking time bomb for protein integrity. *Commun Chem* **2024**, *7*, 1–13.
